# ICGEC: a comparative method for measuring epigenetic conservation of genes via the integrated signal from multiple histone modifications between cell types

**DOI:** 10.1186/s12864-020-6771-1

**Published:** 2020-05-12

**Authors:** Jing Tang, Zefeng Wu, Yuhan Tian, Ruolin Yang

**Affiliations:** College of Life Sciences, Northwest A&F University, Yangling, Shaanxi China

**Keywords:** Epigenetic conservation, Comparative epigenomics, Histone modification, Cell differentiation

## Abstract

**Background:**

Histone post-translational modifications play crucial roles in epigenetic regulation of gene expression and are known to be associated with the phenotypic differences of different cell types. Therefore, it is of fundamental importance to dissect the genes and pathways involved in such a phenotypic variation at the level of epigenetics. However, the existing comparative approaches are largely based on the differences, especially the absolute difference in the levels of individual histone modifications of genes under contrasting conditions. Thus, a method for measuring the overall change in the epigenetic circumstance of each gene underpinned by multiple types of histone modifications between cell types is lacking.

**Results:**

To address this challenge, we developed ICGEC, a new method for estimating the degree of epigenetic conservation of genes between two cell lines. Different from existing comparative methods, ICGEC provides a reliable score for measuring the relative change in the epigenetic context of corresponding gene between two conditions and simultaneously produces a score for each histone mark. The application of ICGEC to the human embryonic stem cell line H1 and four H1-derived cell lines with available epigenomic data for the same 16 types of histone modifications indicated high robustness and reliability of ICGEC. Furthermore, the analysis of the epigenetically dynamic and conserved genes which were defined based on the ICGEC output results demonstrated that ICGEC can deepen our understanding of the biological processes of cell differentiation to overcome the limitations of traditional expression analysis. Specifically, the ICGEC-derived differentiation-direction-specific genes were shown to have putative functions that are well-matched with cell identity. Additionally, H3K79me1 and H3K27ac were found to be the main histone marks accounting for whether an epigenetically dynamic gene was differentially expressed between two cell lines.

**Conclusions:**

The use of ICGEC creates a convenient and robust way to measure the overall epigenetic conservation of individual genes and marks between two conditions. Thus, it provides a basis for exploring the epigenotype-phenotype relationship. ICGEC can be deemed a state-of-the-art method tailored for comparative epigenomic analysis of changes in cell dynamics.

## Background

Gene regulatory networks consist of trans-factors and cis-elements whose interaction with each other governs gene expression dynamics. Conversely, changes in the level and pattern of gene expression might rewire an existing gene regulatory network, leading to cell fate decisions [1], disease development [2] or adaptive evolution of a species [3, 4]. Thus, methods for identifying differentially expressed genes (DEGs) between two conditions of interest have flourished in recent years [5–9]. These methods help to prioritize genes responsible for complex traits and diseases in humans [2] and yield novel insights into cell fate determination [10]. Despite significant progress, an obvious drawback of this type of method is that the molecular mechanism for the differential expression of particular genes remains unidentified.

With the explosive accumulation of histone modification data, the epigenetic mechanisms underlying gene expression dynamics and cell identity have been extensively investigated [11–14]. Histone modifications have been shown to play regulatory roles in hESC differentiation [15, 16] and to be closely related to interspecies differences in gene expression in primates [17, 18]. Although a few studies have disputed the causal effect of histone modifications on gene transcription [19–21], most previous studies have supported the important roles of individual histone modifications in the modulation of transcription [3, 22–24]. Consequently, a general picture has emerged with regard to the combinatorial effects of a few core histone marks: H3K4me3 usually occupies sites in the genome together with H3K27ac, appearing near the transcriptional start sites (TSSs) of active genes [25–27]; both H3K4me1 and H3K27ac frequently occur in the sequences of active enhancers, whereas H3K27me3 and H3K9me3 are characteristic of transcriptional suppression [2, 22, 28, 29]. The availability of dozens of types of histone modification data has also spurred intensive research on the quantitative relationship between gene expression and multiple histone marks via various machine learning methods [30–34], including state-of-the-art deep learning algorithms [35]. In general, these studies indicate that the levels of multiple histone marks can predict the expression levels and even the differential expression of genes [36].

Moreover, some specific types of histone modifications in combination can even instruct the future expression pattern of associated genes. For example, so-called ‘bivalent promoters’ marked with both active H3K4me3 and repressive H3K27me3 marks allow the corresponding genes to be either activated or repressed during the differentiation of hESCs in a manner dependent on future specific developmental signals [37, 38]. Thus, the development of methods that can extract the complex information implied in dozens of histone modifications is particularly important. ChromHMM is one method of this type that enables the chromatin state to be automatically learned from the combinatorial signal of multiple histone marks [12, 39], and the power of ChromHMM has been shown in the study of cellular reprogramming [40]. ChromDiff, which is one of the very few methods focusing on epigenomic comparisons, compares the combinatorial chromatin states between groups of epigenomes [41]. dPCA is another method that compares the epigenomic signal across multiple marks under multiple conditions [42]. Essentially, the two comparative methods utilize either the absolute levels of the raw signals of multiple histone marks or the derived chromatin states thereof to reveal the differentially regulated genes or different regulatory genomic regions between different conditions.

The gene transcriptional state is subject to the control of complex and intricate interplay between multiple histone readers, writers, and erasers [43]. Additionally, the functionality of a gene can be determined by the relative change rather than the absolute levels of its own or partner gene expression. For example, the fold-change detection property of an incoherent feedforward loop is a result of the specific interaction mode between genes, wherein the transcription dynamics of the output gene depend on the relative rather than the absolute change in the input signal [44]. It has been reported that the expression of certain genes in the Wnt [45] and ERK signaling systems [46] is characterized by such properties. Therefore, methods that consider both the relative (rather than the absolute) changes among multiple histone modifications and the pairwise correlations between genes are urgently needed for global epigenome analysis. Specifically, in contrast to traditional methods comparing the absolute epigenomic signals of conspecific or orthologous genes between conditions, an ideal new comparative method should be based on the gene context similarities between two conditions to be compared. Here, the context of a gene is reflected in a vector of the correlation values calculated between that gene and all the other genes with regard to the epigenetic circumstance: the joint signal of multiple histone modifications pertaining to a particular gene. However, to the best of our knowledge, no methods with these features have yet been implemented.

This problem motivated us to propose ‘iterative comparison of gene epigenetic circumstance’ (ICGEC), a new method that utilizes the integrative signal of multiple histone marks to derive gene scores and mark scores for two cell lines to be compared. These scores represent the relative changes in the epigenetic context of corresponding genes and marks, respectively, between two conditions. We elaborate the concept of ICGEC and describe its principle. For the purpose of demonstration, we apply ICGEC to H1 human embryonic stem cells and four cell lines derived from H1 cells with dozens of types of histone modification data in common. Based on the ICGEC-derived scores, we not only confirm the robustness and reliability of ICGEC but also demonstrate that ICGEC indeed provides novel biological insights into the cell differentiation program.

## Results

### Construction of the epigenetic circumstance matrix of genes

**Fig. 1:**
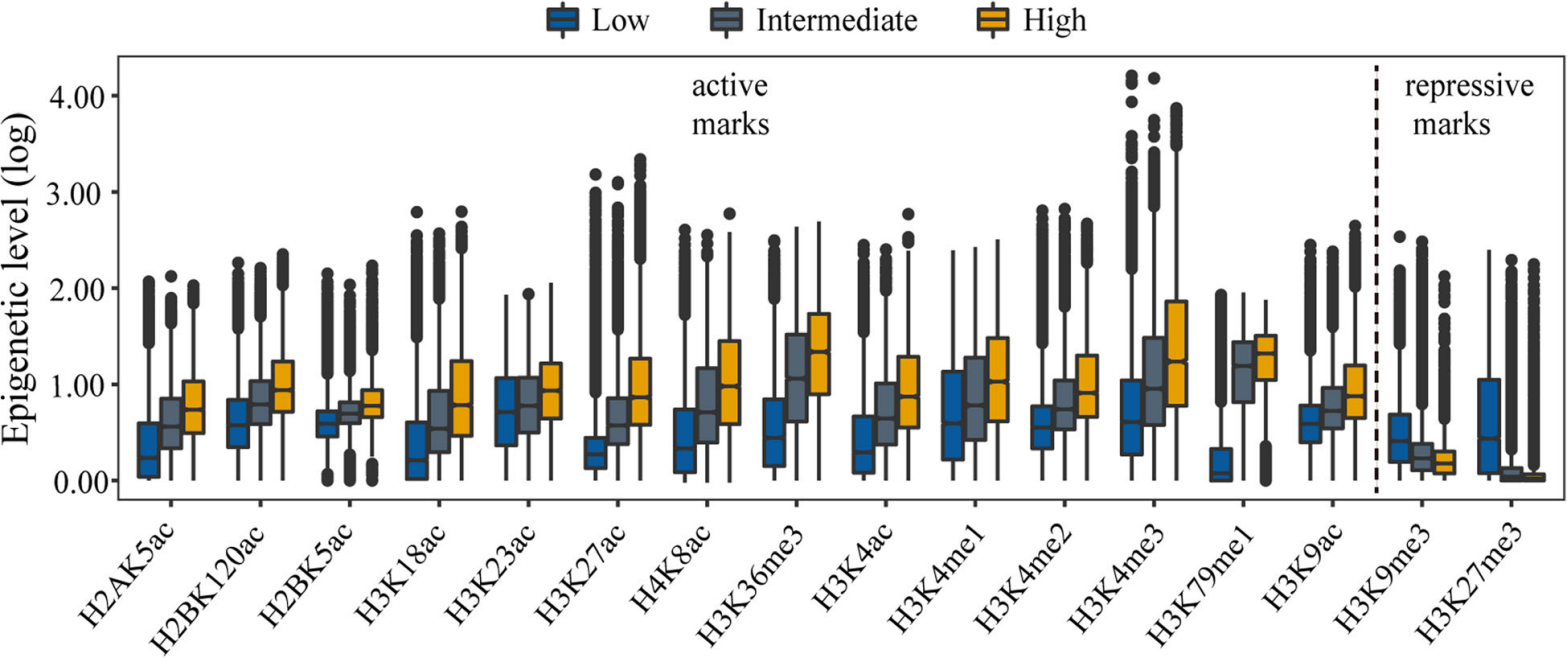
Relationship between gene expression levels and associated epigenetic levels of different histone modifications. Boxplots show the distribution patterns of the epigenetic levels of 16 investigated marks for genes in MSC with low expression (RPKM≤1), intermediate expression (1 < RPKM≤10) and high expression (RPKM > 10)). The levels of histone modifications are from the entire gene locus (Promoter+Body regions). Statistical analysis indicates that the genes with high expression have higher epigenetic levels than the genes with low expression for all except for two repressive marks H3K27me3 and H3K9me3 that display an opposite trend (one-sided Wilcox rank-sum test, *P*-value < 0.05)

To prepare high-quality input data to be used by ICGEC, gene epigenetic circumstance matrices that record the levels of multiple histone marks per gene were produced using a method applied in a recent study [47]. Principally, the multiple histone modification levels of genes were estimated from the signal values of the corresponding peaks within the 2 kb upstream to TSS plus the gene body regions (Promoter+Body) (see Methods). To validate the result, the signal levels of the histone marks were plotted for the human protein-coding genes with high (RPKM> 10), intermediate (1 < RPKM≤10) and low (RPKM≤1) expression levels. As expected, active marks including H3K4me3, H3K36me3, H3K27ac etc., were preferentially present in highly expressed genes, whereas repressive marks, H3K9me3 and H3K27me3, were enriched in genes with low expression (Fig. 1). A similar pattern was observed when the epigenetic signal was computed for promoter and gene body regions, separately (Additional file 1: Figure S1). Together, these results supported the validity of the estimated gene-centric epigenetic levels. Accordingly, we constructed gene epigenetic circumstance matrices for the five cell lines.

### Rationale and principle of ICGEC

Intuitively, the simplest way to find genes underlying cell fate determination, the etiology of diseases and other basic biology at the epigenetic level is to directly compare the absolute levels of each epigenetic mark individually under contrasting conditions, thus revealing genes exhibiting differential modification of particular marks as candidate genes for relevant biological phenomena. Basically, the rationale for this method comes from global gene expression analysis, which focuses on transcriptional behavior instead. Despite its wide application, the expression analysis method shows some limitations because the underlying assumption that the method heavily depends on, i.e., that the cells to be compared synthesize similar amounts of total RNAs, can be violated on some occasions [48]. Therefore, the methodology for addressing epigenetic data might also reveal erroneous genes that are unrelated to a real biological difference. Essentially, the error is caused by the method of the comparison, which focuses on the absolute level of epigenetic modification. To address this issue, we proposed a new comparative method that considers the joint signal of multiple histone modifications rather than individual signals, implicitly utilizes the relative signal among marks and genes and outputs two sets of scores as the estimates of the relative magnitude of the changes with regard to the corresponding epigenetic context of each gene and the gene context of each mark under the two conditions.

**Fig. 2:**
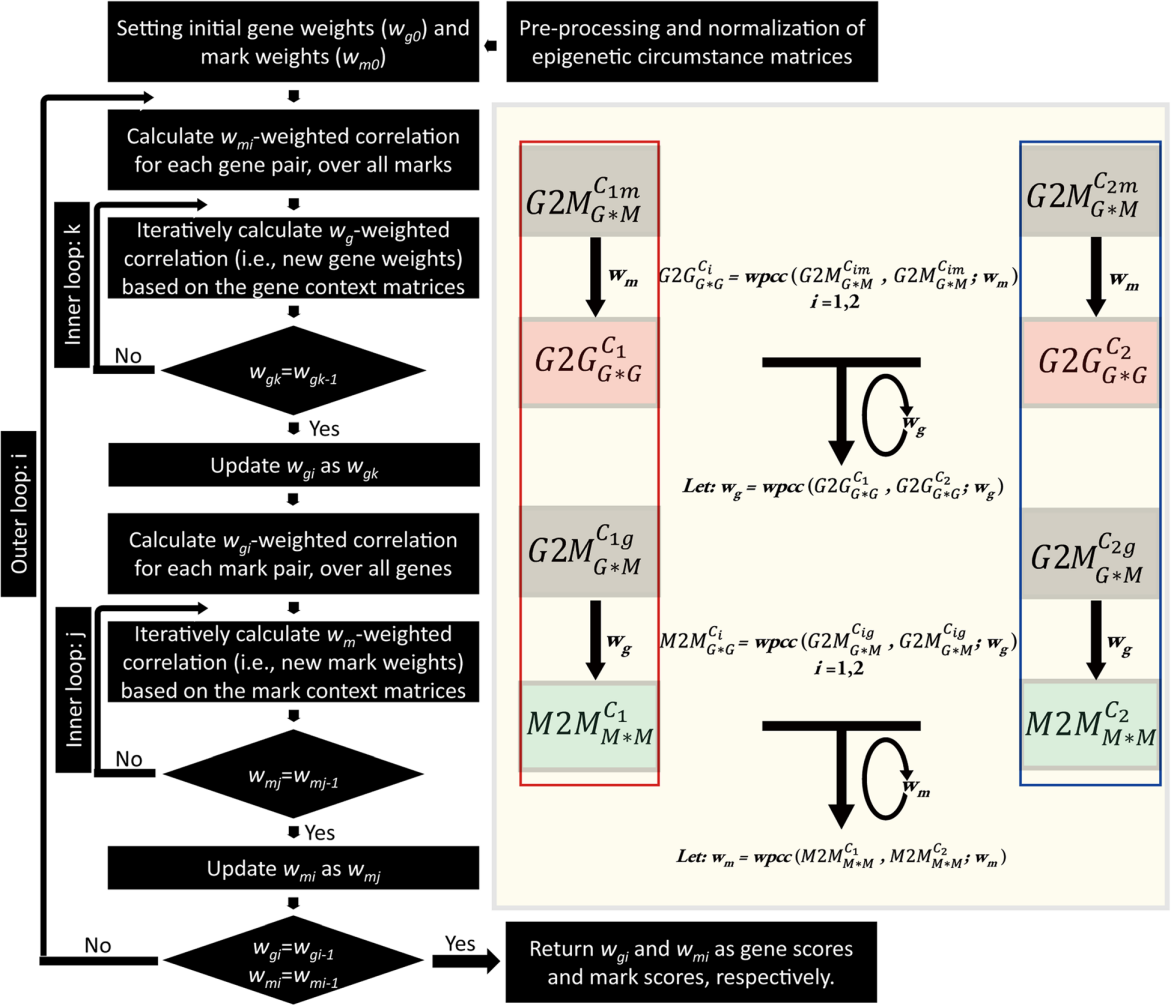
Overall flowchart of ICGEC. The core ICGEC algorithm begins with two normalized matrices ($G2{M}_{G\ast M}^{C_{1m}}$, $G2{M}_{G\ast M}^{C_{2m}}$) from cell lines *C*_*1*_ and *C*_*2*_ to be compared. Then, the *w*_*m*_-based weighted correlation for each gene pair is calculated to produce the context matrices of the genes $G2{G}_{G\ast G}^{C_1}$ and $G2{G}_{G\ast G}^{C_2}$, respectively. Next, the program enters into the 1st inner loop, where *w*_*g*_ is iteratively updated until convergence. Then, the *w*_*g*_-based weighted correlation for each mark pair is calculated to produce the context matrices of the marks ($M2{M}_{M\ast M}^{C_1}$, $M2{M}_{M\ast M}^{C_2}$) from the corresponding normalized matrices $G2{M}_{G\ast M}^{C_{1g}}$ and $G2{M}_{G\ast M}^{C_{2g}}$, respectively. Once it is done, the program enters into the 2nd inner loop, where *w*_*m*_ is iteratively updated until convergence. Finally, the program judges whether the new weights are sufficiently close to the weights in the last round. If yes, the program ends up with *w*_*g*_ and *w*_*c*_ returned as the gene score and mark score, respectively; otherwise, the program enters the outer loop. It should be noted that all of the produced weighted correlations of less than zero must reset to 0 during the iterative process to meet the demands of biological significance. *w*_*g*_ represents gene weights, and *w*_*m*_ represents mark weights. The right panel highlights the calculation process for the two inner loops

Usually, one would estimate the similarities of different genes in terms of their epigenetic circumstances between two conditions with the Pearson correlation coefficients of the pairwise vectors of epigenetic modification levels. The previous method of “iterative comparison of co-expression (ICC)” outperforms the conventional comparison method [49], which weights the conditions in the gene expression profile equally to produce an expression context matrix [50], from which the degree of expression conservation is estimated for all one-to-one orthologous genes between two related species. Inspired by this work, we introduced the concept of epigenetic context, which is quantified for each gene (or mark) as the set of correlation coefficients between a gene (or mark) and all other genes (marks) with regard to the similarities of corresponding epigenetic circumstances. Because different genes and epigenetic marks may exhibit different degrees of conservation across cell lines, leading to unequal contributions to the comparison of the overall epigenetic circumstance, it seems reasonable to give relatively greater weights to those genes or marks with higher conservation. To find the appropriate weights, we adopted a strategy similar to that used in ICC in the search for gene weights. The program iteratively updates the weights until convergence; these weights are referred to as gene scores and mark scores throughout this paper. The workflow of ICGEC is illustrated in Fig. 2. ICGEC can simultaneously estimate the epigenetic conservation of genes and the conservation of histone marks between two cell lines (see Methods). Analogous to previous studies [49, 51], in general, a gene with a larger gene score represents a more stable epigenetic context across cell lines. Ideally, a gene with a gene score extremely close to + 1 means a full conservation of this gene regarding its epigenetic state.

Typical users will use this method to answer questions such as which genes in a cell may experience a substantial change and which genes may be extremely stable in terms of their epigenetic states during development or after a treatment. For this purpose, users will need to collect many types of genome-wide epigenetic data (e.g., histone modifications, DNA methylation, etc.) under the two conditions. Upon applying ICGEC on the data, the results will be provided as a set of scores for each gene and a set of scores for each mark, from which the users would be most interested in those genes or marks showing the greatest changes between the two conditions, which can be interpreted following our example below (refer to Figs. 6a,b and Fig. 7 for details).

### Validation of ICGEC by comparing H1 and MSC cell lines

**Fig. 3:**
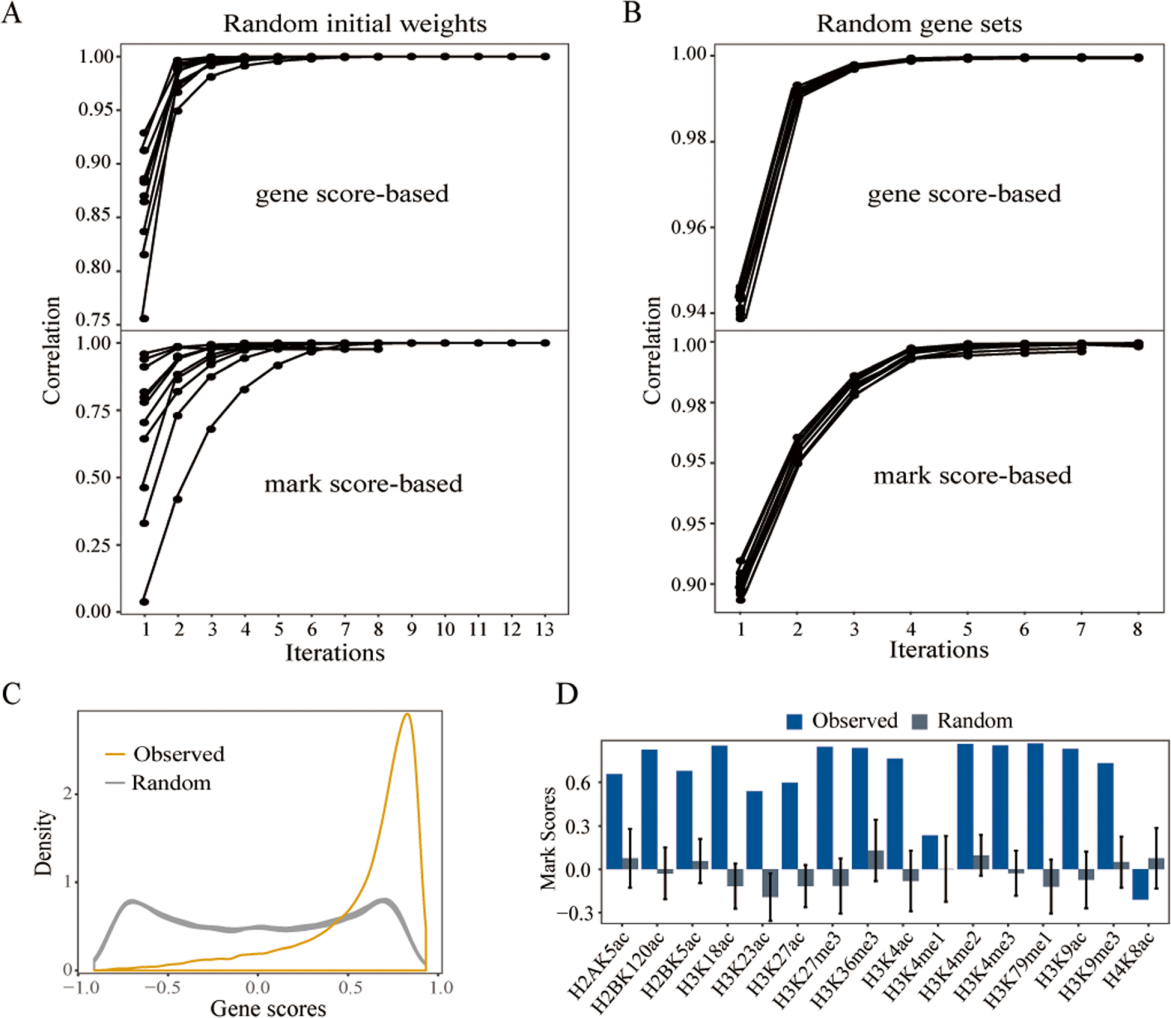
Robustness of ICGEC. **a** Sensitivity of the ICGEC algorithm to random initial weights. ICGEC was run 11 times, one time with equal weights, and the other 10 times with randomly chosen initial weights. The correlation coefficients in regard to the gene scores (upper) and mark scores (lower) between the use of equal weights and random initial weights after 1 to 12 iterations are indicated. **b** Sensitivity of the ICGEC algorithm to a random subset of genes. ICGEC was run 11 times, one time with the full gene sets, and the other 10 times with approximately one-quarter randomly selected genes in the full gene sets. The correlations for gene scores (upper) and mark scores (lower) are indicated between the use of the full and partial data. It should be noted that the correlation coefficients were computed only for the sampled subset of genes. **c** Distributions of gene scores produced with original data (yellow) and permuted data (gray). **d** Mark scores produced with original data (blue) and permuted data (gray). The permuted data used in **c** and **d** were produced by breaking the row equivalence and column equivalence, respectively, of the original two ordered epigenetic circumstance matrices from H1 and MSC by shuffling. ICGEC was run 10 times to produce 10 different permuted datasets, from which the distributions of gene scores and marks scores were generated. The data in **d** are presented as the mean ± sd

To demonstrate the reliability of ICGEC, we applied ICGEC to H1 and MSC cell lines that had available epigenomic data for the same 16 types of histone modifications. First, we ran ICGEC 10 times with random initial weights. In all cases, both the gene scores and mark scores were almost the same as those obtained by using equal initial weights (Fig. 3a). Second, we applied ICGEC to 10 sets of epigenetic circumstance matrices that were downsized by sampling only approximately one quarter of the genes in the full matrices. Consequently, ICGEC converged to the same results (Fig. 3b). Together, the results verified the robustness of ICGEC. Third, we disrupted the correspondence between the genes in the original matrices by permutation. Consequently, rather than peaking around a positive numeric value, the resulting gene scores formed a moderately flat distribution between − 1 and 1 (Fig. 3c). Similarly, we shuffled the histone modification marks, leading to the mark scores being distributed around 0 for most marks (Fig. 3d). Thus, the real gene scores and mark scores were generally greater than would be expected at random, which was in line with the intrinsic link between the two cell lines.

**Fig. 4:**
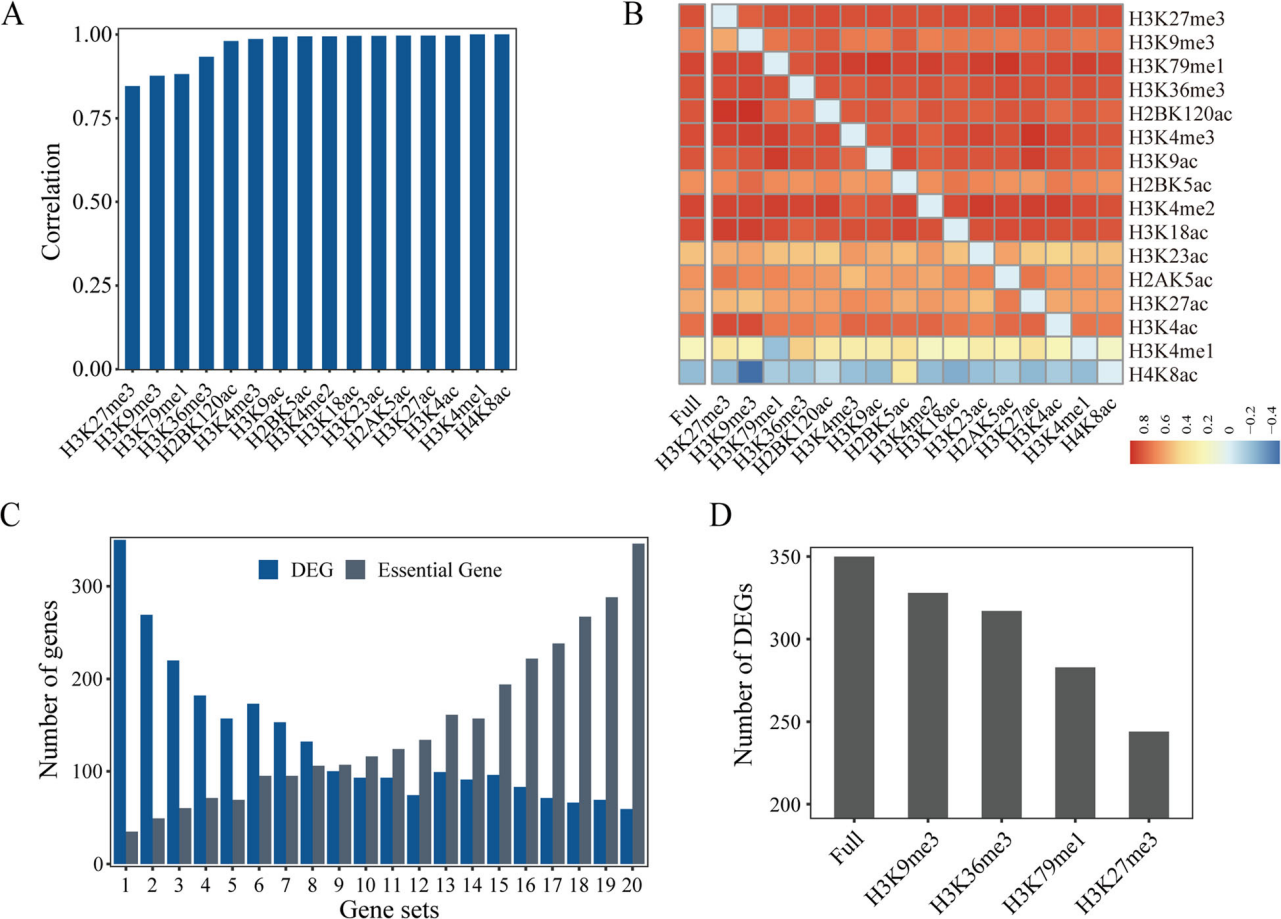
Reliability of ICGEC. **a** Correlation of gene scores between the use of the full data and the leave-one-mark-out data. The labels on the x-axis indicate the individual marks to be removed during ICGEC calculation. **b** Heatmap showing the mark scores obtained with the full data and the leave-one-mark-out data. Each colored cell indicates the mark score of a specific mark (y-axis) produced from datasets with either all marks (“full”) or all but one mark (x-axis). **c** Barplot showing the number of DEGs (blue) and essential genes (gray) included in the gene sets with different levels of epigenetic conservation. The 20 gene sets from left to right presented the lowest to highest gene scores. Genes were classified into bins with proximately equal sizes according to their gene scores. **d** Number of DEGs in the most epigenetically dynamic gene set versus the composition of the mark sets. The labels on the x-axis indicate that either all marks or all but one specific mark were used by ICGEC

Next, we evaluated the relative importance of each histone modification to the overall epigenetic circumstance by running ICGEC on the data removing one mark each time. The greater the difference in the gene scores produced between the use of the full data and the leave-one-mark-out data, the stronger the effect of the mark was on the overall epigenetic circumstance. As indicated by the correlation coefficients, the absence of H3K9me3, H3K27me3, H3K79me1 or H3K36me3 alone led to appreciable changes in the gene scores (Fig. 4a). A confounding factor for the results is the peak type of histone modifications since the four marks are known to have broad peaks in ChIP-Seq data. To clarify this issue, we redid the “one mark removal” analysis using downloaded NarrowPeak data files for the same set of histone marks. Consequently, the peak type seemed to have a limited influence: the top-four marks displaying the largest changes in gene scores (Additional file 1: Figure S2) remained as before, despite that their order has changed. Thus, we demonstrated the indispensable effects of four histone marks on the establishment of the epigenetic circumstance of genes. These results were understandable because these four marks play well-known regulatory roles in gene expression [22, 28, 29, 52]. On the other hand, the gene scores seemed to remain unchanged upon the removal of other marks. In addition, the mark scores were largely unchanged relative to the original values. Together, these results indicated high redundancy of these histone marks contributing to the overall epigenetic circumstance (Fig. 4b). This phenomenon suggests that ICGEC might be robust even when applied to a partial dataset comprising a particular subset of marks from the full data.

Given the close relationship between histone modification and gene expression, we reasoned that the ICGEC-derived gene score that was based on the epigenetic status of genes should reflect the expression dynamics of genes to some extent. As expected, the gene scores were significantly lower for the genes showing at least a two-fold change in expression level than in those without such a change (one-sided Wilcox rank-sum test, *P*-value = 7.57 × 10^− 102^). However, this pattern disappeared for simulated data (Additional file 1: Figure S3). Additionally, when genes were sorted by their gene scores and categorized into 20 equal-sized bins, the numbers of essential genes and DEGs contained in each bin gradually increased and decreased, respectively, with the increase in gene scores (Fig. 4c). Furthermore, the removal of the abovementioned four marks resulted in a different degree of reduction in the number of DEGs in the bin with the lowest gene scores (Fig. 4d), providing indirect evidence that these histone marks act as important regulators of gene expression [30]. Taken together, the results showed that ICGEC is reliable in that the ICGEC-derived scores reflect biologically significant changes in the epigenetic circumstance of genes and to some extent mirror gene expression dynamics.

### ICGEC can reveal cell differentiation-related biological processes

**Fig. 5:**
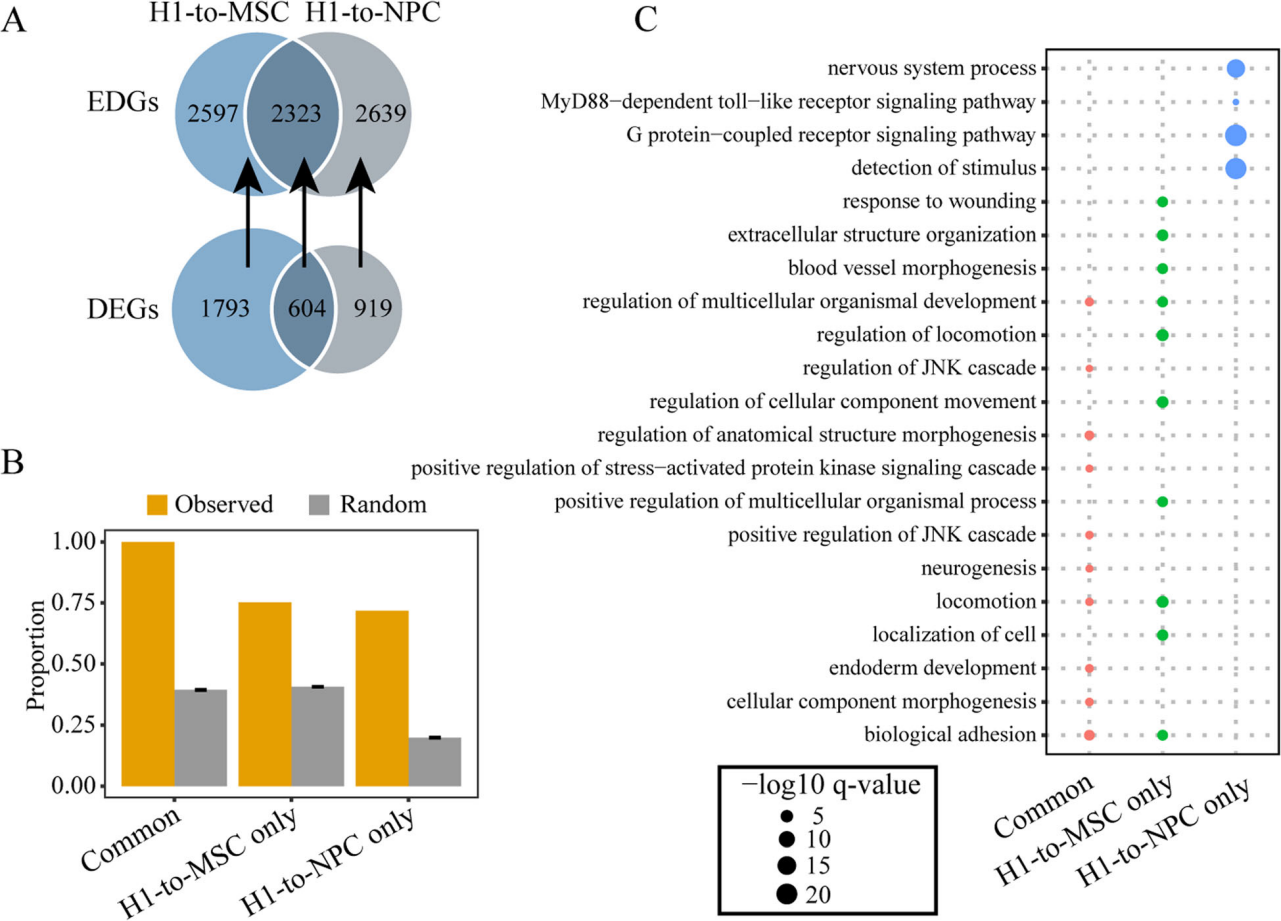
Significant correspondence between epigenetic dynamics and expression dynamics. **a** Venn diagrams displaying the number of common and direction-specific EDGs and DEGs during differentiation from H1 to MSC or NPC, respectively. The arrows indicate the three gene set pairs to be tested for the degree of gene overlap. **b** Bar plot showing the proportions of DEGs in the corresponding EDGs. These proportions from observed data were clearly significantly (*P* < 0.001) different from those from randomly permutated data. The simulated results are presented as the mean ± sd. **c** Bubble chart showing the GO terms enriched for the three sets of EDGs. Dot sizes are scaled to the enrichment significance

Triggered by various developmental cues, the human H1 cell line can be differentiated into different derived cell lines. Considering two developmental programs of cell fate determination, some genes may experience similar changes in expression in both differentiation directions, while some other genes may undergo an expression shift in only one specific direction. The same rules apply to the epigenetic status of genes. Therefore, an interesting question concerns the correspondence between genes showing alterations in their epigenetic circumstances and genes showing dynamic expression. To explore this issue and to demonstrate the potential biological significance that ICGEC may reveal, we performed the following comparative analysis. We divided the genes into four equal groups: the genes in the group with the lowest gene scores were defined as epigenetically dynamic genes (EDGs), whereas the genes in the group with the highest gene scores were defined as epigenetically conserved genes (ECGs). Focusing on two specific differentiation processes, 1793 and 919 genes were only found to be differentially expressed from H1 to MSC and H1 to NPC, respectively, while 604 genes displayed significant expression changes in both directions. In turn, 2597, 2639 and 2323 corresponding EDGs were identified (Fig. 5a). Using a permutation test (see Methods), we found that the DEGs in the “common”, “H1-to-MSC-only” and “H1-to-NPC only” groups significantly overlapped with these corresponding EDGs (Fig. 5b). Intriguingly, significant overlap was also observed between ECGs and DEGs, but the degree of overlap was lower than that between DEGs and EDGs, as expected (Additional file 1: Figure S4A, B). We performed the same analysis by randomly selecting two other pairs of derived cell lines. As a result, similar patterns were obtained (Additional file 1: Figure S4C-F).

The EDGs assigned to the three categories allowed us to further elucidate the biological processes associated with the alteration of the epigenetic circumstance in only one or two differentiation directions. Remarkably, the EDGs identified only in the differentiation from H1 to MSC (H1-to-MSC-only) were enriched in biological processes related to terms such as “regulation of locomotion”, “positive regulation of multicellular organismal process”, “regulation of cellular component movement”, “extracellular structure organization”, and “blood vessel morphogenesis”, which seemed to be concordant with the physiology of MSC. In contrast, the EDGs in the “H1-to-NPC-only” group were enriched in the “G protein-coupled receptor signaling pathway” and “nervous system process” terms, which seemed to match the identity of NPC quite well (Fig. 5c). However, biological process terms such as “cellular component morphogenesis”, “regulation of anatomical structure morphogenesis”, “positive regulation of JNK cascade”, “endoderm development” and “cellular component morphogenesis” were only enriched for the EDGs in the “common” group. Therefore, these results indicated that the genes sorted by ICGEC based on the change in the epigenetic state can provide a reasonable explanation for expression dynamics during cell differentiation, at least from the perspective of epigenetics.

Overall, we not only demonstrated the epigenetic mechanisms underlying the dynamic expression of genes involved in cell fate decisions but also verified that ICGEC is useful for identifying biologically significant genes and processes during cell development.

### ICGEC can identify differentiation direction-specific signatures

**Fig. 6:**
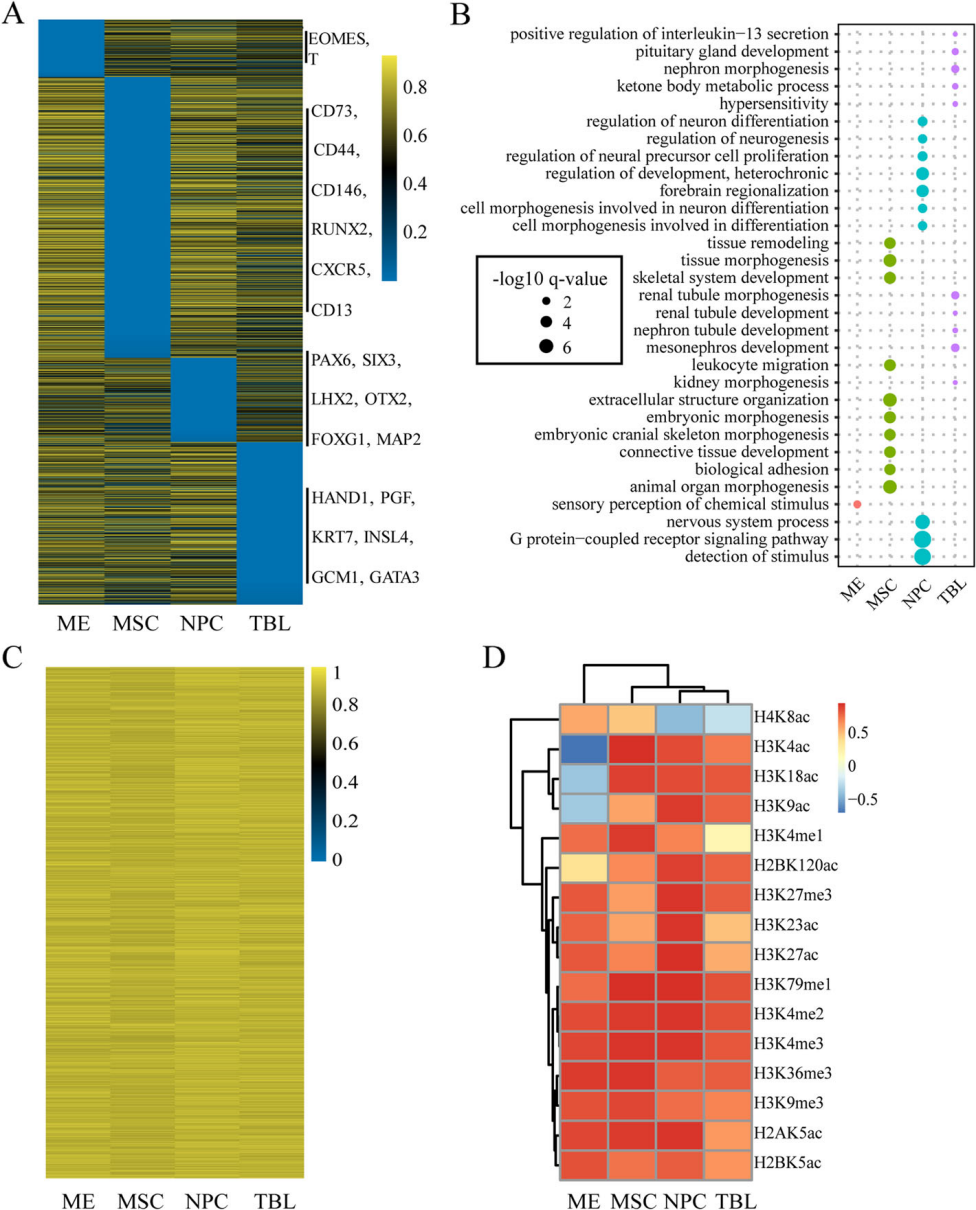
Characterization of DDSGs and DDUGs. **a** Heatmap showing the gene scores of DDSGs. Genes are organized by the cell line in which the epigenetic conservation is lowest across the four developmental directions. Known mark genes defined by expression pattern that happen to be DDSGs are shown on the right. **b** Bubble chart displaying the overrepresented GO terms for DDSGs. **c** Heatmap showing the gene scores of DDUGs. **d** Comparison of mark scores for each histone mark across the four differentiation directions. The data are clustered for better representation. The labels on the x-axis in (**a-d**) represent the specific derived cell line-associated differentiation directions

Cell line-specific genes are critical determinants of cell identity. Therefore, it is fundamental to study differentiation direction-specific genes. Traditionally, these genes are determined by their expression patterns, and such genes are collectively referred to as “marks”. To obtain more comprehensive insight into the diversity of the differentiation programs from H1 to different derived cell lines, we screened the so-called “marks” that corresponded to the four specific differentiation directions based on the epigenetic dynamics rather than the expression patterns. Using the entropy-based method [13, 53, 54], 200, 543, 938 and 282 epigenetically dynamic differentiation-direction-specific genes (DDSGs) were identified that displayed significant changes in their epigenetic circumstance during the differentiation of H1 into a specific derived cell line (Fig. 6a). Strikingly, the known cell line-specific marks determined by expression were mostly recovered from the four sets of DDSGs (Fig. 6a). Furthermore, biological processes related to stem cell maintenance and differentiation were enriched for the DDSGs. For example, the DDSGs corresponding to the differentiation from H1 to MSC were enriched in “extracellular structure organization”, “animal organ morphogenesis”, “tissue morphogenesis”, “skeletal system development” and “connective tissue development”, which appeared to be highly consistent with the characteristics of MSC [55, 56] (Fig. 6b). The DDSGs associated with TBL were overrepresented in terms such as “pituitary gland development” and “nephron morphogenesis”. In parallel, DDSGs that corresponded to the other two differentiation directions were enriched in related biological processes (Fig. 6b). Additionally, we determined 745 epigenetically conserved differentiation-direction-ubiquitous genes (DDUGs) that exhibited consistently high epigenetic conservation across the four differentiation processes (Fig. 6c). As expected, essential genes were enriched in the DDUGs (Hypergeometric test, *P*-value = 5.57 × 10^− 90^). Therefore, the results indicated that ICGEC enables the reliable identification of differentiation direction-specific signatures.

Particular genes may undergo significant changes during the cell differentiation process with regard to the epigenetic circumstance; on the other hand, some specific types of histone modifications may undergo relatively large changes during the same process. As shown in Fig. 6d, regardless of the absolute magnitude of the mark scores for different histone modifications, histone acetylation marks such as H4K8ac, H3K4ac, H3K18ac and H3K9ac displayed considerable variation across different differentiation directions, which suggested that these marks may play important but distinct roles in different developmental trajectories. In a sense, this result was compatible with the observation that global deacetylation of histones is required during stem cell differentiation [57, 58]. In summary, ICGEC provides a basis for a comprehensive comparative analysis across multiple cell lines based on the integrated epigenetic data of genes, thereby revealing novel biological insights.

### DEGs and non-DEGs within EDGs exhibit distinct biological significance

**Fig. 7:**
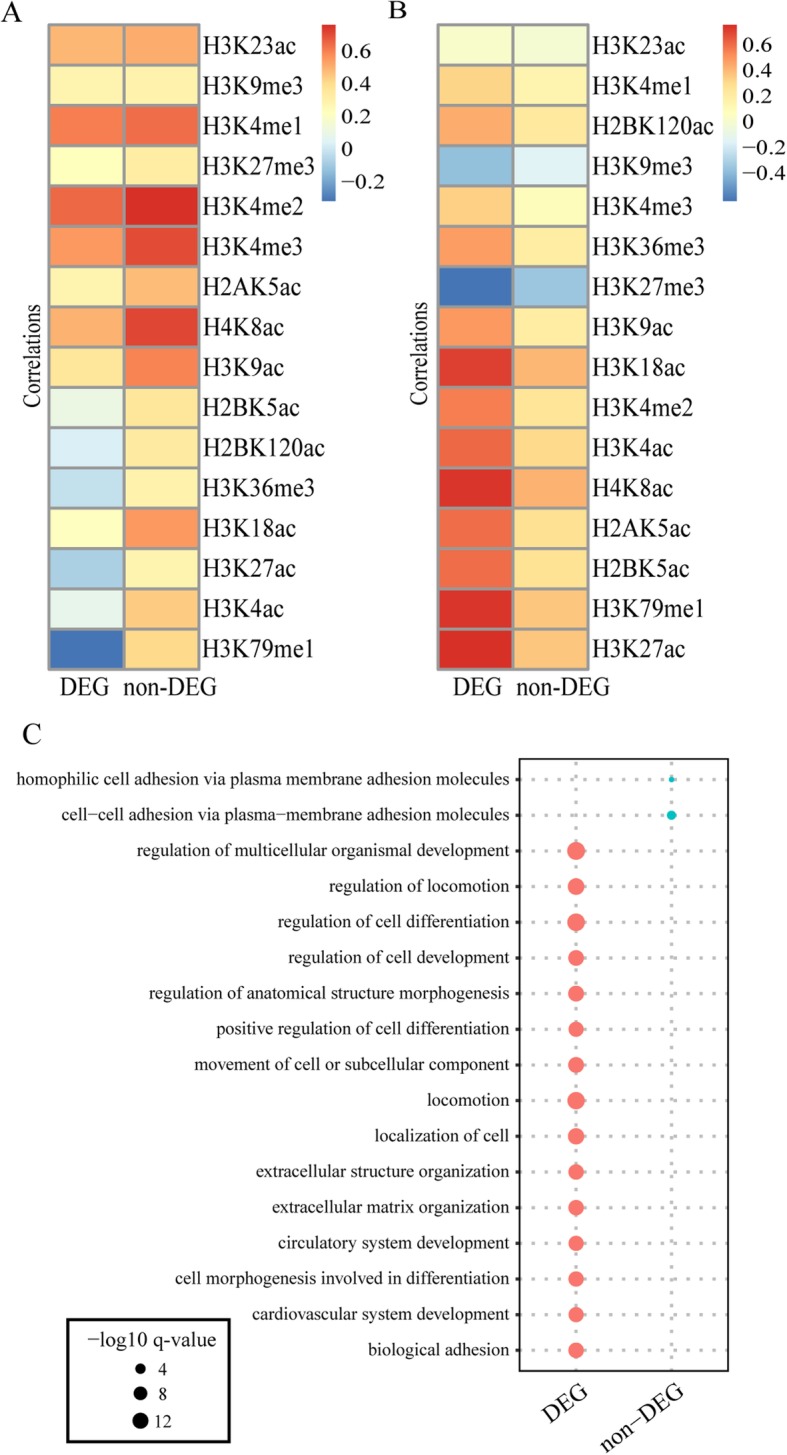
Distinct epigenetic signatures and biological functions between DEGs and non-DEGs among EDGs. **a** Pearson correlations in terms of the histone modification levels between H1 and MSC for DEGs and non-DEGs. **b** Pearson correlations in terms of the expression changes and epigenetic changes for each mark for DEG and non-DEGs. Here, the expression change was calculated for DEGs and non-DEGs as the difference in the expression levels of the corresponding genes divided by the sum between H1 and MSC. Similarly, the epigenetic changes for each mark were calculated. The marks in (A-B) are positioned by the difference in the correlations between DEGs and non-DEGs in ascending order. **c** Bubble chart showing different GO terms enriched for the DEGs and non-DEGs

Although the EDGs included disproportionately high numbers of DEGs, they also included a considerable number of non-differentially expressed genes (non-DEGs) that exhibited higher gene scores than the DEGs among the EDGs on average (Additional file 1: Figure S5). We sought to understand why some of the EDGs were DEGs while others were not between two cell lines. Considering that the ICGEC just provides a measure of overall epigenetic conservation of genes and marks, we speculate that the different expression dynamics of the DEGs and the non-DEGs might be related to the difference in the influence degree of the different marks on the two sets of genes. To explore this possibility, we simply compared the DEGs and the non-DEGs with regard to the dynamics of each mark acting on them. Specifically, we calculated the Pearson correlation coefficient of each histone modification between before and after the cell differentiation of H1 for the DEGs and the non-DEGs, separately. When the marks were sorted by the difference in the similarity between DEGs and non-DEGs, H3K79me1 and H3K27ac stood out, as they always exhibited relatively large differences across the four differentiation directions (Fig. 7a and Additional file 1: Figure S6A-C). Additionally, for most marks, such similarities were smaller for the DEGs, indicating that the epigenetic circumstance was more dynamic in the DEGs than in the non-DEGs. Furthermore, we related the changes in gene expression to the changes in the marks between two cell lines to be compared for the DEGs and the non-DEGs separately. Consequently, all but two repressive marks, H3K27me3 and H3K9me3, were found to be positively associated with gene expression to varying degrees, which was highly consistent with the known functions of these marks in gene regulation. It was no coincidence that H3K79me1 and H3K27ac were among the few marks showing the greatest reduction in correlation between the expression changes and epigenetic changes in DEGs relative to those in non-DEGs across the four differentiation directions (Fig. 7b and Additional file 1: Figure S6D-F), as they have well-documented regulatory functions in gene transcription [59, 60]. The overall conclusions hold when using the Spearman correlation, which is robust to extreme values and outliers, although the exact order of marks has changed as compared with the results of using the Pearson correlation (Additional file 1: Figure S7).

In addition, GO enrichment analysis clearly distinguished the DEGs from the non-DEGS identified between H1 and MSC: the former were significantly associated with terms such as “regulation of multicellular organismal development”, “regulation of cell differentiation”, “regulation of anatomical structure morphogenesis” and “regulation of cell development”, while the latter were enriched in terms such as “cell-cell adhesion via plasma-membrane adhesion molecules” (Fig. 7c). In addition, TFBS enrichment analysis indicated that the DEGs and non-DEGs were preferentially targeted by distinct TFs: the former were collectively regulated by dozens of TFs with diverse functions, such as TFs related to the cell cycle (E2F family, TFDP1), early development (SP, EGR family) and cellular growth (NRF1), whereas the latter were favored by a muscle differentiation-related TF, Myog (Additional file 1: Figure S8). Therefore, the ICGEC-derived results in combination with differential expression analysis may provide a basis for understanding the distinct molecular functions of DEGs and non-DEGs among EDGs during cell fate determination.

Overall, our in-depth analysis not only provided evidence that the different marks may contribute to the altered epigenetic circumstances between the DEGs and non-DEGs but also indicated that the ICGEC-derived results provide a starting point from which genes with distinct expression dynamics and accompanying biological functions can be identified.

## Discussion

In this study, we proposed ICGEC, a novel method for quantifying the relative degree of epigenetic conservation of genes and marks between two cell lines. Although ICGEC will become ICC if all the marks are given the same weights throughout the process of computation, it is quite different from ICC, which only characterizes genes in terms of their epigenetic dynamics, as ICGEC can simultaneously assess the conservation of histone marks. To the best of our knowledge, no other methods developed thus far exhibit this characteristic. Interestingly, although ICC implicitly employs equal mark weights rather than more appropriate weights such as those used in ICGEC, a primary analysis of H1 and MSC indicated that ICC yields similar outcomes to ICGEC in terms of gene scores (data not shown). This seems to be understandable, as reflected by the relatively high and similar ICGEC-derived mark scores for almost all except for one or two marks (Fig. 3d). However, we consider it a coincidence because the two methods are designed for addressing different challenges. In order to solve the difficulty of cross-species gene expression comparison that the transcriptomes to be compared are unusually from different set of conditions, making it infeasible to directly compare the expression data from two species, ICC was proposed to compare the expression context rather than the expression profiles. Its essence is to compares the architecture of the co-expression networks. While ICGEC, bearing in mind that different epigenetic marks contribute to gene regulation in varying degrees, was proposed to evaluate the epigenetic conservation for both genes and marks between two cell types of a same species. Therefore, the two algorithms applying to a same data would result in different results regarding the conservation of genes only if a large variation is existed among the marks regarding their contribution to the overall epigenetic context. A great advantage of ICGEC over ICC is that ICGEC is able to determine the relative extent to which a mark might play a major or minor role in maintaining the epigenetic circumstance of genes across two cell lines genome wide. Most importantly, as illustrated in Fig. 7 and Additional file 1: Figure S6A-C, by jointly analyzing gene expression and the ICGEC-derived results, we can reveal the epigenetic mechanism underlying the different expression patterns of different sets of genes between two conditions, which may improve our understanding of gene transcription regulation.

During implementation, the ICGEC algorithm utilizes two context matrices, $G2{G}_{G\ast G}^C$ and $M2{M}_{M\ast M}^C$, both of which are derived from *G*2*M*^*C*^ and used for estimating the similarity of the epigenetic context between corresponding genes and marks, respectively, under two conditions. Basically, we could produce a similar estimate based on the epigenetic circumstance by directly comparing the *G*2*M*^*C*1^ and *G*2*M*^*C*2^ of two cell lines. This means that we implicitly consider the architecture of the gene regulatory network equivalently under the two conditions and admit that the raw epigenetic signal values are comparable. However, in many cases, the raw data from two conditions may be ill matched; they could be produced by different experimental techniques or different laboratories. Therefore, a transformation operation is necessary, which ensures the reliability of ICGEC.

The negative ICGEC scores observed in this study are harder to interpret in a biological context than the non-negative scores because it is generally easy for us to accept that a gene with a score very close to 1 indicates that gene with a near perfect epigenetic conservation and a gene with a score around 0 indicates the gene with a very low conservation, but it is more difficult to understand the negative score. So it is appealing to implement a new scoring system limited by [0,1]. The simplest way to achieve the goal is to perform a linear transformation on the original scores. However, a serious defect of such kind of transformation is that the real difference of the original scores derived from different comparisons is masked. In fact, the negative score can be understood according to how the ICGEC scores are produced. Basically, the scores are the weighted PCCs of two big vectors corresponding to the epigenetic context of two conditions. So theoretically, the scores can be any values between − 1 and + 1. However, due to the intrinsic biological connection between the cells to be compared, for a vast majority of genes, their gene scores are positive, only a very small number of genes being negative. In contrast, when the biological relationship is broken or weakened, the scores shift to the side of lower values: the number of genes with negative scores will increase, but the number of genes with positive scores will decrease (Fig. 3c and Additional file 1: Figure S9). So ideally, if the raw data matrices from two closely related cell lines have no noise, the genes with a full conservation should get a score of + 1, while the genes with the lowest conservation should get a score close to 0. However, in reality there exist noise from various sources, so that a small number of genes would inevitably get numerically negative ICGEC scores. In our view, the epigenetic status of these gene has substantially changed just like those genes with gene scores around 0.

We evaluated the relative importance of individual marks to the overall epigenetic circumstance using “mark removal” analysis. In essence, unary, binary, ternary, quaternary and any plausible *n*-ary mark removal analyses could be performed to systematically explore the effects of any higher-order combinations of marks on the gene scores. This kind of analysis would be useful to identify those marks as a whole that their combined functions are currently unknown but they indeed play a potential role in gene regulation via a yet unknown complex functional interplay, thereby spurring new hypotheses. In addition, the “mark removal” analysis also revealed the high redundancy among the marks, suggesting that the results might be robust even when applying ICGEC to some particular subsets of marks. When data collection is technically difficult or costly for some marks, it becomes especially important for a method, like ICGEC, capable of using a subset of marks to produce results comparable to those obtained with full datasets. An ensuing question is how to find the subset of marks. A straightforward method is to perform a series of *n*-ary mark removal analysis to evaluate the equivalence between all possible sets of marks based on the change in gene score. However, it should be pointed out that there are also some caveats for using only a subset of marks. First, it would be impossible to find a unique subset of marks. For example, supposing seven marks A-G are under study, if the removal analysis indicates that mark A represents an equivalent contribution as mark B, mark C in combination with mark D is equivalent to the combination of marks E, F and G, then four schemes (marks A, C and D; marks A, E, F and G; marks B, C and D; marks B, E, F and G) are valid with regard to the subsets. Second, the larger statistical noise during the course of PCC calculation resulting from using less marks might be further exaggerated due to the iterative process of ICGEC, leading to a worse result. Third, although many studies have revealed that a few “core” marks are sufficient to predict gene expression, other marks can still exert their influence to gene regulation, probably acting on a smaller set of genes and/or in a more subtle manner. In a word, running ICGEC with less marks seems to provide a reasonable alternative solution to the same question, but it must be used carefully, particularly in the situation that only a few marks are involved in the analysis.

Given the close relationship between gene expression and epigenetic modifications, one might think that a gene with a significant change in gene expression level should experience a significant change of its epigenetic status. Thus, it seems counterintuitive that the ECGs had a significant overlap with the DEGs. While research progress in the past decade has proved that gene expression is subjected to a complex and multi-layered regulation [61]. Besides histone post-translational modification, DNA modification, DNA accessibility, nucleosome occupation and spatial topology of chromosomes have played important roles in gene expression regulation. Therefore, ICGEC offers a new opportunity to distinguish these different mechanisms for the change in gene expression level.

In this study, we applied ICGEC to the epigenomic data of the human embryonic stem cell line H1 and four cell lines derived from H1. The reason for choosing these data was that the raw data were produced under strict experimental curation and processed via a unified analysis pipeline. In addition, the full datasets have been subjected to extensive comparative epigenomic analysis [13] and are frequently used as good data resources for exploring other questions [15, 52, 62, 63]. While it must be noted that ICGEC can be used with imperfectly-matched data or data with low to moderate levels of noise (Additional file 1: Figure S9) and that it is not limited to the analysis of gene-centric metrics, ICGEC can also be applied to the epigenomic data of noncoding regions and extended for cross-species comparison. A specific application of ICGEC is to create genome-wide tracks of conservation for marks between two cell types given the appropriate data. Basically, this can be accomplished in the following way. First, the genome can be divided into 1 Mb-sized windows. Second, for each window, 1000 non-overlapping regions of 1 kb are defined, and their associated epigenetic levels for various marks can then be estimated. Third, ICGEC is applied to calculate the overall conservation of each mark in each 1 Mb window. Lastly, the genome-wide conservation can be visualized for every mark.

In this study, the set of genes that were categorized as epigenetically dynamic according to the ICGEC-derived gene scores but were not identified as differentially expressed caught our attention. Although DNA methylation is always a potential factor related to gene expression alteration [64], we sought to understand those phenomena that may be related to or at least partially caused by the changes in epigenetic circumstances underpinned by dozens of types of histone modifications. Through detailed comparative analysis of DEGs versus non-DEGs among the EDGs, we revealed that a few marks, including H3K79me1 and H3K27ac, with well-known functions in gene transcription regulation might be involved in this process (Fig. 7a and b). In addition, we showed that in striking contrast to the DEGs favored by TFs related to processes such as the cell cycle, early development and cellular growth, the non-DEGs among the EDGs were preferentially targeted by TFs involved in muscle differentiation (Additional file 1: Figure S8). Coincidentally, MSC are committed to differentiate into muscle, adipose, bone, cartilage and connective tissues during development [56]. Therefore, we speculate with caution that the epigenetically dynamic but non-differentially expressed genes might indicate a poised state in which these genes would be activated in response to an indicator of the next developmental stage. Strikingly, the non-DEGs exhibited lower expression than the DEGs on average in MSC (one-sided Wilcox rank-sum test, *P*-value = 1.20 × 10^− 93^). Correspondingly, the levels of both active H3K4me3 and repressive H3K27me3 were higher in the non-DEGs than in the DEGs (one-sided Wilcox rank-sum test, P-value =1.12 × 10^− 5^ and 3.80 × 10^− 3^, respectively), which is reminiscent of the concept of a “bivalent promoter” [38]. The identification of the exact function of these non-DEGs and the molecular mechanism underlying their transcriptional behavior awaits further experimental verification.

So far, the results presented in the main text are based on the epigenetic signal from the entire gene locus (Promoter+Body). One might wonder whether the other way of quantifying the signals might influence the ICGEC results. To investigate this issue, we applied ICGEC to the data of H1 and MSC, where the epigenetic signal was recomputed by three other ways: 1) from the promoter region alone; 2) from the gene body region alone; and 3) from promoter and gene body regions, separately. Obviously, as shown from the similarity of gene scores, the method of quantifying the epigenetic signal indeed has influenced the ICGEC results (Additional file 1: Figure S10). It seems that the “Promoter+Body” scheme produces gene scores very similar to that from the “Body only” scheme, but differs with the “Promoter only” scheme to the largest degree (Additional file 1: Figure S10A). We also found a small variation regarding the proportions of DEGs in EDGs among the four schemes, whereas a relative large difference was observed for the proportions of essential genes in ECGs (Additional file 1: Figure S10B, C). In our view, it makes sense that the epigenetically conserved gene set should be enriched with essential genes, while the epigenetically dynamics gene set should be depleted with essential genes. Clearly, the “Promoter+Body” scheme outperforms the alternatives in this respect.

In this work, only the binary information of TF binding was used for the TFBS enrichment test. Actually, adjacent TF binding sites for the same TF species (homotypic clusters) are prevalent in the promoters and enhancers of humans and other organisms [65]. Also, it is suggested that the specific organization of homotypic clusters can modulate the temporal dynamics of TF binding through multiple physical mechanisms, thereby influencing gene expression [66]. Therefore, the enrichment analysis used here seems a bit simplistic. However, if we distinguish one occurrence of a TFBS from multiple occurrence, it may introduce additional noise because the identification of cis-regulatory module in which homotypic clusters reside is a pretty complex process. Therefore, we chose the simple method to compare the DEGs and non-DEGs within EDGs. Interestingly, our primary analysis showed that for almost all the TF that preferentially target the DEGs or non-DEGs, their target genes contained in the DEGs or non-DEGs indeed had statistically more TFBS instances in their promoters than the targets within the whole protein-coding genes (data not shown).

A common problem in cross-species gene expression analysis is that the ill-match of expression datasets causes a large difference in the distribution of gene-gene correlation coefficients between different conspecific and allospecific datasets. To overcome this problem, Guan et al. proposed a metric, local network similarity (LNS) to quantify expression divergence of orthologous genes [67]. A merit of LNS is that it explicitly adjusts the variance of the distribution of within- and between-species gene-gene correlation coefficients by adopting a two-step normalization strategy: using the Fisher transformation to transfer the correlation coefficients firstly and then normalizing these data to the standard normal distribution. We compared two versions of ICGEC, either embedding with the Guan’s normalization procedure or without. As a result, the ICGEC scores were almost unchanged (the PCCs for gene score and mark score are 0.987 and 0.963, respectively). It is understandable because it seems that the two distributions of gene-gene correlation coefficients differed slightly, though statistically significant (Additional file 1: Figure S11). We advise the users to run ICGEC with Guan’s normalization into account if the distribution of the correlation coefficients data represents a large difference between the two conditions.

## Conclusions

We proposed a new method, ICGEC, which provides a convenient and robust way to measure the overall epigenetic conservation of individual genes and marks to be investigated between two conditions. As exemplified by the analysis of the basic process of human embryonic stem cell differentiation, we demonstrated that ICGEC, whether used alone or in combination with traditional expression analysis, can provide novel biological insights. Importantly, it is easy for us to apply ICGEC to other genomic entities such as the genome-wide DNA-hypersensitive sites, given that the relevant epigenetic features are correctly calculated. In addition, ICGEC can be flexibly used for between-species comparisons. Therefore, this method can be deemed a general method tailored for comparative epigenomic analysis.

## Methods

### Data collection

The original histone modification data and transcriptome data from the H1 human embryonic stem cells and four cell lines derived from them (mesendoderm cells (ME), trophoblast-like cells (TBL), mesenchymal stem cells (MSC), neural progenitor cells (NPC)) were produced by the NIH Roadmap Epigenomics Mapping Consortium [11]. In this study, sixteen types of histone modifications, including H2AK5ac, H2BK120ac, H2BK5ac, H3K18ac, H3K23ac, H3K27ac, H3K27me3, H3K36me3, H3K4ac, H3K4me1, H3K4me2, H3K4me3, H3K79me1, H3K9ac, H3K9me3 and H4K8ac, were included in the whole analysis because they were available for all five cell lines. For these marks, downloaded processed data files in BroadPeak format were used to produce the epigenetic circumstance matrix. For the transcriptome data produced by RNA-Seq, we downloaded a unified data (RPKM) file and reads mapping (BAM) files for different purposes. The download links to these datasets analyzed in this study are shown in Additional file 2: Table S1 and S2.

A total of 3316 human essential genes were retrieved from the database of essential genes (essentialgene.org/) [68].

### Construction of the epigenetic circumstance matrix of genes

By reference to a recently published paper [47], the signal intensity of a mark on each gene (i.e., the level of histone modification) was calculated as the weighted sum of the peak signal values over all peaks within a specified genomic region. Here the peak signal value denotes the average intensity of each histone modification peak, which was directly retrieved from the downloaded BroadPeak format files. Basically, this calculation can be represented by ${\sum}_{p=1}^n{L}_p\times {S}_p/{L}_g$, where *n* is the total peak number of a specific histone modification that are either completely located within or partially overlapped with an entire gene locus between 2 kb upstream to the TSS and the TTS of this gene *g*; *L*_*p*_ is the length of a peak overlapping with the genomic region; *L*_*g*_ denotes the length of the whole region of focus on a gene *g*; and *S*_*p*_ is the peak signal value. Accordingly, five epigenetic circumstance matrices that corresponded to the respective cell lines were constructed. It must be noted that there are other methods of quantifying the epigenetic level of genes. For example, the signal intensity of a mark can be estimated from the promoters alone, from the gene body regions alone or from the promoter and gene body regions, separately.

### ICGEC method

#### Preprocessing and normalization of raw epigenetic data matrices

First, for two cell lines, *C*_*1*_ and *C*_*2*_, to be compared by ICGEC, their associated epigenetic circumstance matrices, $G2{M}^{C_1}$ and $G2{M}^{C_2}$, were ordered to ensure that the equivalent rows corresponded to the epigenetic circumstances of the same genes and that the equivalent columns corresponded to the epigenetic levels of all genes for the same types of marks in the two cell lines. Next, the matrices were preprocessed and normalized as follows. First, the genes in which at least half of the histone modifications presented zero signals in both matrices were discarded. Second, the resultant matrices were logarithmically transformed. Third, two matrices, $G2{M}_{G\ast M}^{C_{1m}}$ and $G2{M}_{G\ast M}^{C_{2m}}$, were produced, which were scaled by marks from the logarithmic matrices. Fourth, two additional matrices, $G2{M}_{G\ast M}^{C_{1g}}$ and $G2{M}_{G\ast M}^{C_{2g}}$, were produced, which were scaled by genes from $G2{M}_{G\ast M}^{C_{1m}}$ and $G2{M}_{G\ast M}^{C_{2m}}$, respectively. Accordingly, four normalized matrices were prepared to be directly used by ICGEC. The normalization procedure ensures that the resultant matrices exhibit zero mean and unit variance with respect to the marks and genes in each cell line, allowing a meaningful comparison of the same genes and marks between two conditions through their associated gene profiles and mark profiles, respectively [69].

#### Iterative calculation of gene scores and mark scores

In general, the main program of ICGEC is comprised of one outer loop and two inner loops, which are organized into five main steps.

In the first step, the epigenetic context matrices $G2{G}_{G\ast G}^{C_1}$ and $G2{G}_{G\ast G}^{C_2}$ were derived from $G2{M}_{G\ast M}^{C_{1m}}$ and $G2{M}_{G\ast M}^{C_{2m}}$, respectively, by calculating the weighted Pearson correlation coefficients (*wPCCs*) between every pair of genes using the wtd.cors function implemented in the R package ‘weights’ [70]. This step represents the entry of the outer loop. The first time through the loop, identical initial weights of all marks were given.

Second, *wPCCs* were calculated for equivalent rows in $G2{G}_{G\ast G}^{C_1}$ and $G2{G}_{G\ast G}^{C_2}$, which corresponded to the estimates of similarity between the epigenetic context of the corresponding genes under two conditions. Identical initial weights of all genes were used. This step represents the entry of the first inner loop. Through the inner loop, new gene weights are returned, which will be used thereafter in the next cycle until convergence.

Third, the matrices $G2{M}_{G\ast M}^{C_{1g}}$ and $G2{M}_{G\ast M}^{C_{2g}}$ were converted into $M2{M}_{M\ast M}^{C_1}$ and $M2{M}_{M\ast M}^{C_2}$, respectively, by calculating the *wPCCs* between every pair of marks. The weights of the genes used herein were obtained from the above inner loop.

Fourth, we manipulated the matrices $M2{M}_{M\ast M}^{C_1}$ and $M2{M}_{M\ast M}^{C_2}$ in the same way as in the second step to obtain mark weights instead of gene weights. Through this second inner loop, the mark weights were updated until convergence. Additionally, identical initial mark weights were used.

Last, the main program judges whether the weights that were just returned from the two inner loops presented sufficiently small differences from their predecessors. If yes, the program will end with the gene weights and mark weights returned; at this time point, these weights are called gene scores and mark scores. If no, the program will return to the first step and iteratively update the weights until convergence. ICGEC is robust to the initial gene weights and mark weights.

### Identification of differentially expressed genes

The differentially expressed genes (DEGs) between H1 and the four derived cell lines were identified using Cufflinks (Version 2.2.1) [71]. Specifically, the DEGs were defined as genes showing at least two-fold changes in expression levels and an FDR < 0.01. In total, 3425, 6262, 6968 and 5062 DEGs were obtained between H1 and ME, TBL, MSC and NPC, respectively.

### Permutation test

To reveal whether the genes with altered epigenetic circumstances and the genes showing dynamic expression exhibited significant overlap, the observed number of DEGs identified in only one or two differentiation directions in the corresponding EDGs was compared with random expectation. The significance level was estimated using a permutation test. Specifically, for the DEGs identified in either differentiation direction, we randomly repartitioned them into three sets with the gene numbers equal to the sizes of corresponding observed DEG sets, and the number of genes in the simulated datasets that overlapped with the corresponding EDGs was counted. This process was repeated 1000 times, and an empirical *P* value was derived by comparing the actual values with the simulated data.

### Gene ontology (GO) analysis

ToppGene [72] was used to identify enriched GO terms in the gene sets of interest using default parameters. The reference gene set was all of the genes included in the matrices under comparison. The overrepresented GO terms (Benjamini-Hochberg multiple test correction: q-value < 0.05) with respect to the “Biological Process” subontology were identified.

### TFBS enrichment analysis

The potential transcription factor binding sites (TFBS) in the genomic regions of 500 bp around the TSSs of the target genes were scanned using FIMO with default parameters according to the PWMs of 572 known TFs [73]. The PWMs were downloaded from the JASPAR database (http://jaspar.genereg.net/downloads/). For each TF, the hypergeometric test was used to determine whether a gene set was preferentially regulated by the TF relative to all of the genes addressed by ICGEC.

### Identification of epigenetically dynamic differentiation-direction-specific genes

To identify epigenetically dynamic differentiation-direction-specific genes (DDSGs), we used Shannon entropy-based method [13, 53, 54]. Specifically, all negative gene scores were reset to 1.0 × 10^− 6^, and the entropy of each gene with regard to its four ICGEC-derived gene scores resulting from the comparisons between H1 and the four derived cell lines was then computed. Next, the DDSGs were further refined from the genes with the bottom 20% entropy values. We required that the gene score of a DDSG in the associated differentiation direction was at least 2.5 times lower than in other directions and no more than the 5% quantile of the entire gene score. Additionally, epigenetically conserved differentiation-direction-ubiquitous genes (DDUGs) were selected from the top 20% of genes, whose gene scores were greater than the 80% quantile of the entire gene score.

## Supplementary information

**Additional file 1: Figure S1.** Relationship between gene expression levels and epigenetic levels estimated from promoter and gene body regions for different histone modifications. Boxplots show the distribution patterns of the levels of 16 investigated marks from gene body (upper panel) or promoter (lower panel) regions for genes in MSC with low expression (RPKM≤1), intermediate expression (1 < RPKM≤10) and high expression (RPKM > 10)). Statistical analysis indicates that the genes with high expression have higher epigenetic levels than the genes with low expression for all except for two repressive marks H3K27me3 and H3K9me3 that display an opposite trend (one-sided Wilcox rank-sum test, *P*-value < 0.05). **Figure S2.** Reliability of ICGEC on using the NarrowPeak data. (**A**) Correlation of gene scores between the use of the full data and the leave-one-mark-out data. The labels on the x-axis indicate the individual marks to be removed during ICGEC calculation. (**B**) Heatmap showing the mark scores obtained with the full data and the leave-one-mark-out data. Each colored cell indicates the mark score of a specific mark (y-axis) produced from datasets with either all marks (“full”) or all but one mark (x-axis). **Figure S3.** Comparison of the gene scores between genes showing at least a two-fold change in expression level versus those without such a change. Boxplots showing that the stably expressed genes (|log2FC| ≤ 1) have a higher gene scores than the dynamically expressed genes (|log2FC| > 1) between H1 and MSC. However, no such pattern was observed for permuted data (right). **Figure S4.** Correspondence between genes showing alterations in the epigenetic circumstances and genes showing dynamic expression. (**A**) Venn diagrams displaying the number of common and direction-specific ECGs and DEGs during differentiation from H1 to MSC or NPC, respectively. The arrows indicate the three gene set pairs to be tested for the degree of gene overlap. (**B**) Bar plot showing that the proportions of DEGs in the corresponding ECGs are significantly higher than those from using randomly permutated data for the comparison from H1 to MSC or NPC. The simulated results are presented as the mean ± sd. (**C**-**D**) Bar plots showing that the proportions of DEGs in the corresponding EDGs are significantly higher than those from using randomly permutated data for the for the comparison from H1 to MSC or NPC and for the comparison from H1 to MSC or TBL, respectively. (**E**-**F**) Bar plots showing that the proportions of DEGs in the corresponding ECGs are significantly higher than those from using randomly permutated data for the comparison from H1 to MSC or NPC and for the comparison from H1 to MSC or TBL, respectively. **Figure S5.** Comparison of the gene scores between DEG and non-EDG among EDGs. Boxplots showing that the gene scores of DEGs among EDGs are significantly lower than those of non-DEGs among EDGs for the comparison between H1 and MSC. **Figure S6.** (**A-C**) Pearson correlations in terms of the histone modification levels between H1 and ME, between H1 and TBL, and between H1 and NPC, respectively, for DEGs and non-DEGs, respectively. (**D-F**) Pearson correlations in terms of the expression changes and epigenetic changes for each mark between H1 and ME, between H1 and TBL, and between H1 and NPC, respectively, for DEG and non-DEGs. The similar method of creating Fig. 7a was used to calculate the expression changes and epigenetic changes for each mark for the DEGs and non-DEGs. The marks in (A-F) are positioned by the difference in the correlations between DEGs and non-DEGs in ascending order. **Figure S7.** (**A-D**) Spearman correlations in terms of the histone modification levels between H1 and MSC, between H1 and ME, between H1 and TBL, and between H1 and NPC, respectively, for DEGs and non-DEGs, respectively. (**E-H**) Spearman correlations in terms of the expression changes and epigenetic changes for each mark between H1 and MSC, between H1 and ME, between H1 and TBL, and between H1 and NPC, respectively, for DEG and non-DEGs. The similar method of creating Fig. 7a was used to calculate the expression changes and epigenetic changes for each mark for the DEGs and non-DEGs. The marks in (A-H) are positioned by the difference in the correlations between DEGs and non-DEGs in ascending order. **Figure S8.** TFs that preferentially bind to DEGs and non-DEGs among EDGs, respectively, between H1 and MSC. The bar length represents the significance level of q-value (in logarithmic scale). **Figure S9.** Effect of additional noise on the performance of ICGEC. **(A)** Density plot showing the shift of the gene score produced from datasets without (s = 0) or with (s = 0.2, 0.4, 0.6, 0.8 and 1.0) additional artificial noise. **(B)** Hierarchical clustering plot showing the similarity of gene scores produced at different noise levels. The similarity is estimated as PCC over all genes with regard to their gene scores. The arrow indicates the dataset without additional noise. **(C-D)** Heatmaps showing the overlap degree of EDGs **(C)** and ECGs **(D)**, respectively, identified between using datasets with additional noise at five levels and without. The EDGs or ECGs identified at the five noise levels were divided into 20 bins of approximately equal size, then the number of genes that were present at the equivalent bins corresponding to the dataset without additional noise were counted. For this part of analysis, we began with the epigenetic data matrices (s = 0) from the H1 and MSC cell lines, then we added different levels of additional noise to both matrices to produce artificially less perfect datasets, finally we evaluate the performance of ICGEC based on the results shown here. The added noise was derived from the distribution U (− 1, 1) × s × e, where s represented the noise strength from low to high with corresponding values from 0.2 to 1 with a step size of 0.2, e was the original modification level, and U (− 1,1) denoted a uniform distribution from − 1 to 1. Our results indicate that ICGEC performs well in despite of low-to-moderate level of noise. **Figure S10.** Comparison of ICGEC using epigenetic signal data from four methods. **(A)** Pair-wise Pearson correlation coefficient of ICGEC gene scores derived from using the four methods that quantify the epigenetic signal of genes from the promoter alone (Promoter), gene body regions alone (Body), from the promoter and gene body regions (Promoter+Body), and from the promoter and gene body regions, separately (Promoter+Body (32)). **(B, C)** Bar plot showing the number of DEGs **(B)** and essential genes **(C)** in EDGs or ECGs, respectively. The genes with ICGEC gene scores on the top and bottom one-quarter of all genes were defined as epigenetically dynamic genes (EDGs) and epigenetically conserved genes (ECGs), respectively. **Figure S11.** Comparison of the distributions of gene-gene correlation coefficients between H1 and MSC cell lines. Density plot showing the distribution pattern of gene-gene correlation coefficients, which were calculated from the gene epigenetic context matrices for H1 and MSC cell lines, separately. Kolmogorov-Smirnov test indicates that two distributions are significantly (*P*-value < 0.05) different from each other though the two density plots seem to be similar to some extent.

**Additional file 2: Table S1.** The download links to the histone modification data used in this study. **Table S2.** The download links to the RNA sequencing data used in this study.

## Data Availability

The download links to the histone modification data and RNA sequencing data used in this study can be found in Additional file 2. The source code of ICGEC can be accessed at https://github.com/FionaTJ/ICGEC.

## Abbreviations

DDSG: Epigenetically dynamic differentiation-direction-specific gene
DDUG: Epigenetically conserved differentiation-direction-ubiquitous gene
DEG: Differentially expressed gene
ECG: Epigenetically conserved gene
EDG: Epigenetically dynamic gene
FC: Fold change
FDR: False discovery rate
GO: Gene ontology
ICC: Iterative comparison of co-expression
ICGEC: Iterative comparison of gene epigenetic circumstance
PWM: Position weight matrix
RPKM: Reads per kilobase per million mapped reads
TFBS: Transcription factor binding site
TSS: Transcription start site
TTS: Transcription termination site
