# Supplementary material for: ICGEC: a comparative method for measuring epigenetic conservation of genes via the integrated signal from multiple histone modifications between cell types
Source: BMC Genomics. 2020 May 12;21:356. doi: 10.1186/s12864-020-6771-1 (PMC7216622; doi:10.1186/s12864-020-6771-1)
Supplement: Supplementary file 1 — Additional file 1: Figure S1. Relationship between gene expression levels and epigenetic levels estimated from promoter and gene body regions for different histone modifications. Boxplots show the distribution patterns of the levels of 16 investigated marks from gene body (upper panel) or promoter (lower panel) regions for genes in MSC with low expression (RPKM≤1), intermediate expression (1 < RPKM≤10) and high expression (RPKM > 10)). Statistical analysis indicates that the genes with high expression have higher epigenetic levels than the genes with low expression for all except for two repressive marks H3K27me3 and H3K9me3 that display an opposite trend (one-sided Wilcox rank-sum test, P-value < 0.05). Figure S2. Reliability of ICGEC on using the NarrowPeak data. (A) Correlation of gene scores between the use of the full data and the leave-one-mark-out data. The labels on the x-axis indicate the individual marks to be removed during ICGEC calculation. (B) Heatmap showing the mark scores obtained with the full data and the leave-one-mark-out data. Each colored cell indicates the mark score of a specific mark (y-axis) produced from datasets with either all marks (“full”) or all but one mark (x-axis). Figure S3. Comparison of the gene scores between genes showing at least a two-fold change in expression level versus those without such a change. Boxplots showing that the stably expressed genes (|log2FC| ≤ 1) have a higher gene scores than the dynamically expressed genes (|log2FC| > 1) between H1 and MSC. However, no such pattern was observed for permuted data (right). Figure S4. Correspondence between genes showing alterations in the epigenetic circumstances and genes showing dynamic expression. (A) Venn diagrams displaying the number of common and direction-specific ECGs and DEGs during differentiation from H1 to MSC or NPC, respectively. The arrows indicate the three gene set pairs to be tested for the degree of gene overlap. (B) Bar plot showing that the proportion [file 12864_2020_6771_MOESM1_ESM.docx]

Supplementary Material

**ICGEC: a comparative method for measuring epigenetic conservation of genes via the integrated signal from multiple histone modifications between cell types**

This supplementary material file includes:

Figures S1-S11

**
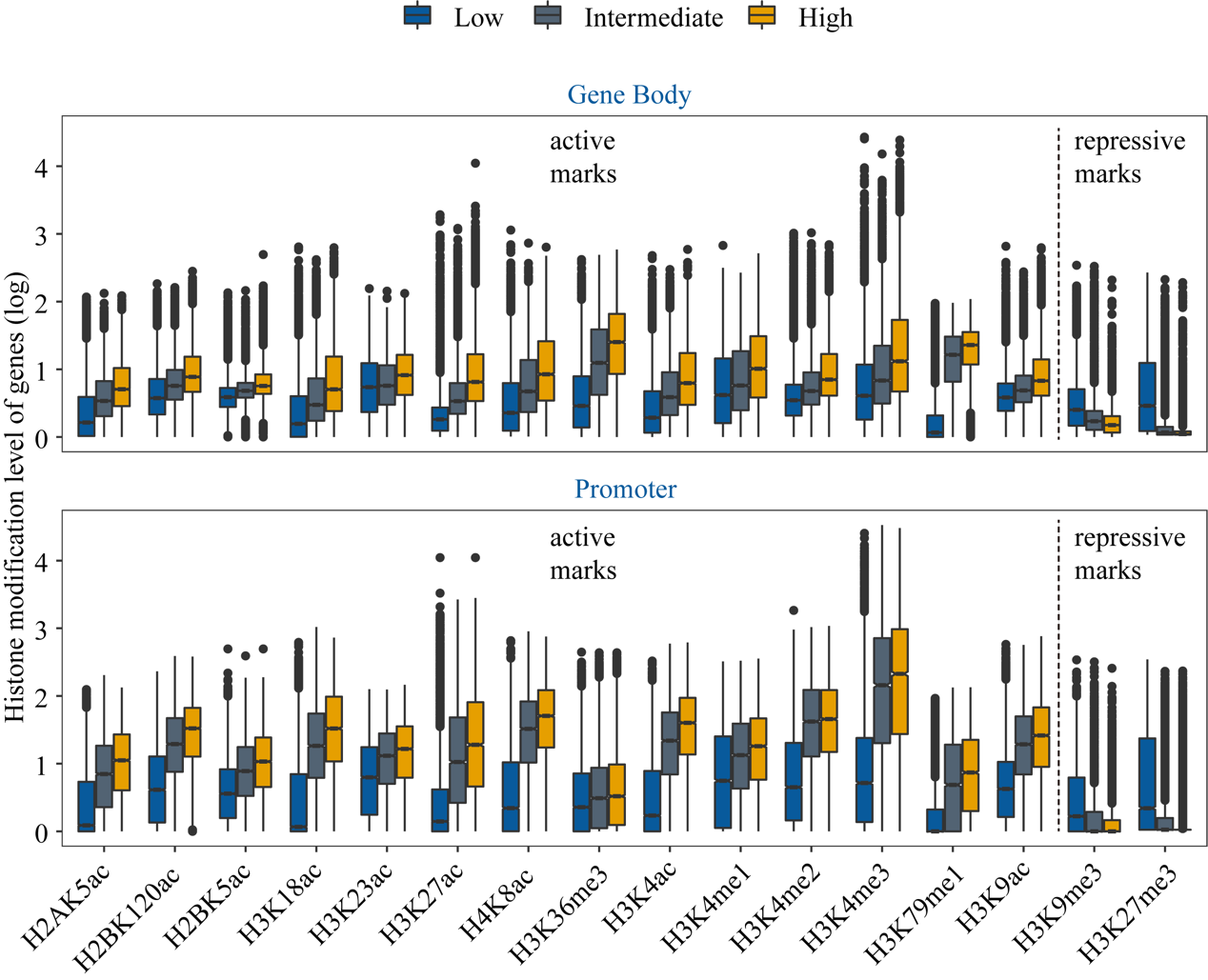
**

**Figure S1 Relationship between gene expression levels and epigenetic levels** **estimated from promoter and gene body regions for different histone modifications.** Boxplots show the distribution patterns of the levels of 16 investigated marks from gene body (upper panel) or promoter (lower panel) regions for genes in MSC with low expression (RPKM≤1), intermediate expression (1<RPKM≤10) and high expression (RPKM >10)). Statistical analysis indicates that the genes with high expression have higher epigenetic levels than the genes with low expression for all except for two repressive marks H3K27me3 and H3K9me3 that display an opposite trend (one-sided Wilcox rank-sum test, P-value < 0.05).


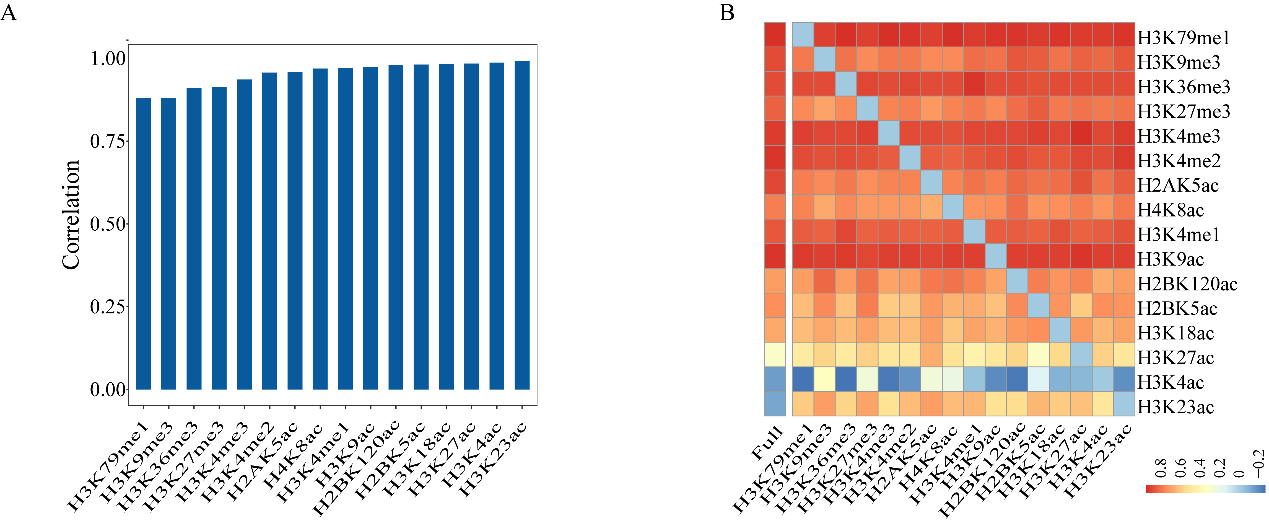


**Figure S2 Reliability of ICGEC on using the NarrowPeak data.** (**A**) Correlation of gene scores between the use of the full data and the leave-one-mark-out data. The labels on the x-axis indicate the individual marks to be removed during ICGEC calculation. (**B**) Heatmap showing the mark scores obtained with the full data and the leave-one-mark-out data. Each colored cell indicates the mark score of a specific mark (y-axis) produced from datasets with either all marks (“full”) or all but one mark (x-axis).


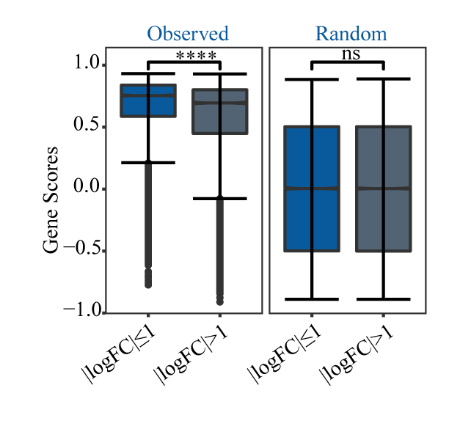


**Figure S3 Comparison of the gene scores between genes showing at least a two-fold change in expression level versus those without such a change.** Boxplots showing that the stably expressed genes (|log2FC|≤1) have a higher gene scores than the dynamically expressed genes (|log2FC|>1) between H1 and MSC. However, no such pattern was observed for permuted data (right).


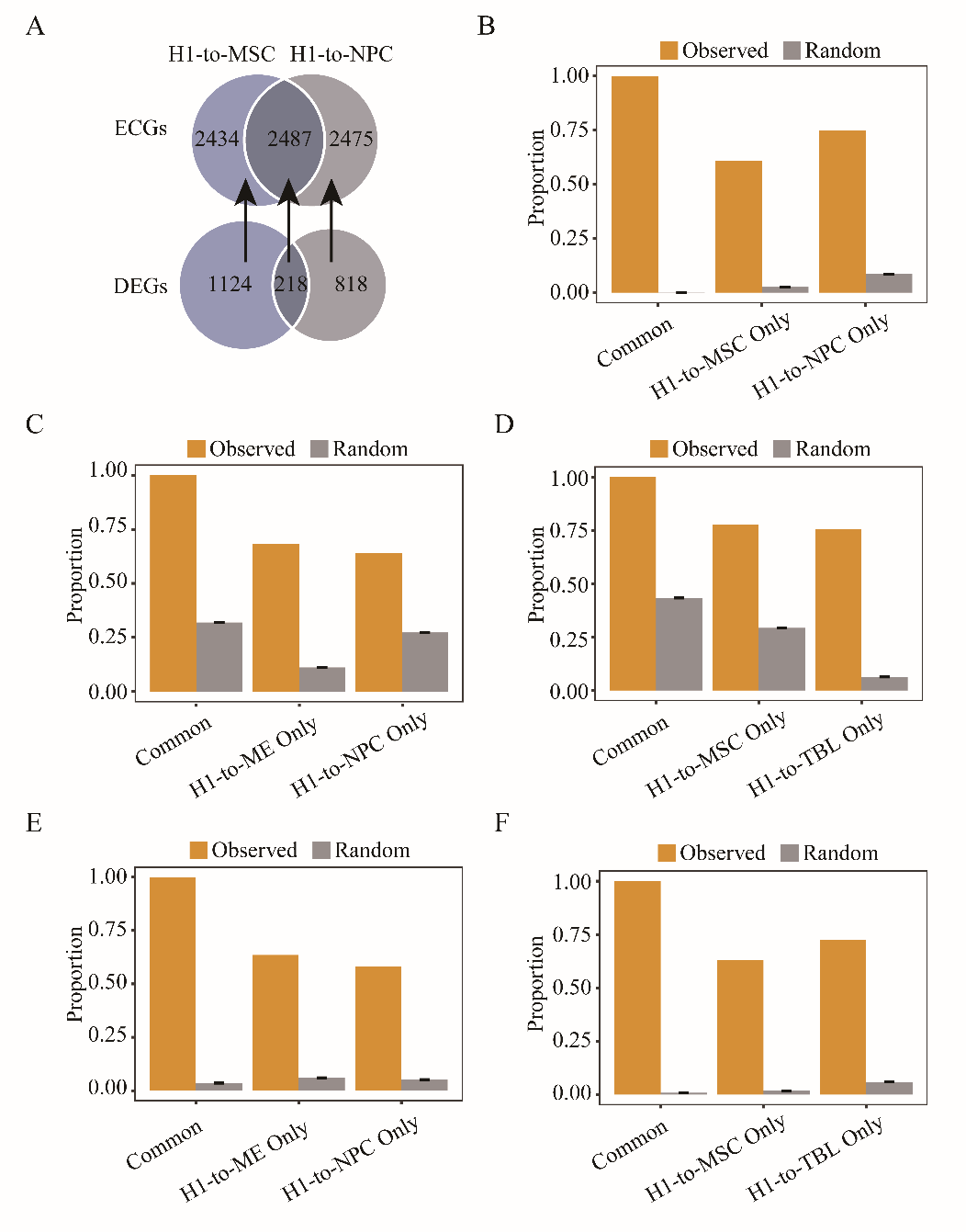


**Figure S****4** **Correspondence between genes showing alterations in the epigenetic circumstances and genes showing dynamic expression.** (**A**) Venn diagrams displaying the number of common and direction-specific ECGs and DEGs during differentiation from H1 to MSC or NPC, respectively. The arrows indicate the three gene set pairs to be tested for the degree of gene overlap. (**B**) Bar plot showing that the proportions of DEGs in the corresponding ECGs are significantly higher than those from using randomly permutated data for the comparison from H1 to MSC or NPC. The simulated results are presented as the mean ± sd. (**C**-**D**) Bar plots showing that the proportions of DEGs in the corresponding EDGs are significantly higher than those from using randomly permutated data for the for the comparison from H1 to MSC or NPC and for the comparison from H1 to MSC or TBL, respectively. (**E**-**F**) Bar plots showing that the proportions of DEGs in the corresponding ECGs are significantly higher than those from using randomly permutated data for the comparison from H1 to MSC or NPC and for the comparison from H1 to MSC or TBL, respectively.


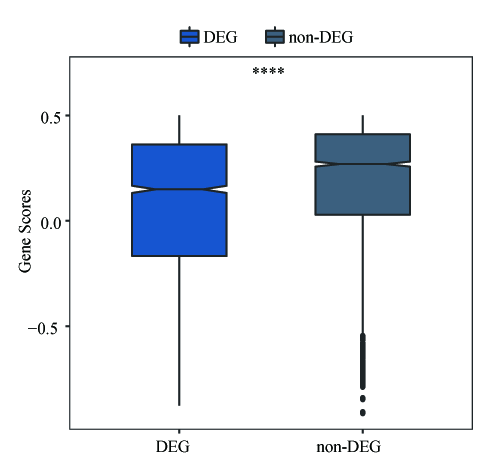


**Figure S5 Comparison of the gene scores between DEG and non-EDG among EDGs.** Boxplots showing that the gene scores of DEGs among EDGs are significantly lower than those of non-DEGs among EDGs for the comparison between H1 and MSC.


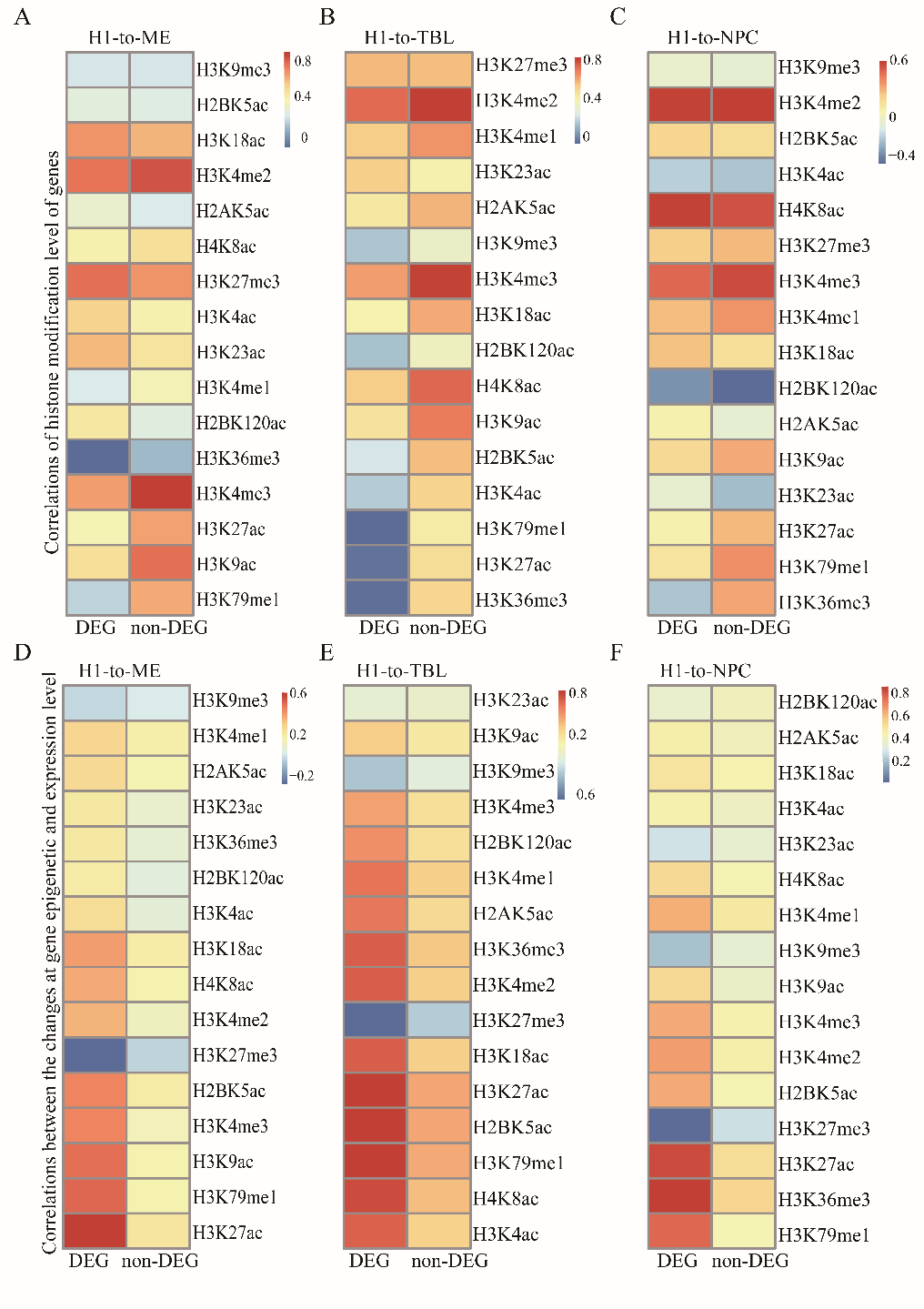


**Figure S****6** (**A-C**) Pearson correlations in terms of the histone modification levels between H1 and ME, between H1 and TBL, and between H1 and NPC, respectively, for DEGs and non-DEGs, respectively. (**D-F**) Pearson correlations in terms of the expression changes and epigenetic changes for each mark between H1 and ME, between H1 and TBL, and between H1 and NPC, respectively, for DEG and non-DEGs. The similar method of creating figure 7A was used to calculate the expression changes and epigenetic changes for each mark for the DEGs and non-DEGs. The marks in (A-F) are positioned by the difference in the correlations between DEGs and non-DEGs in ascending order.


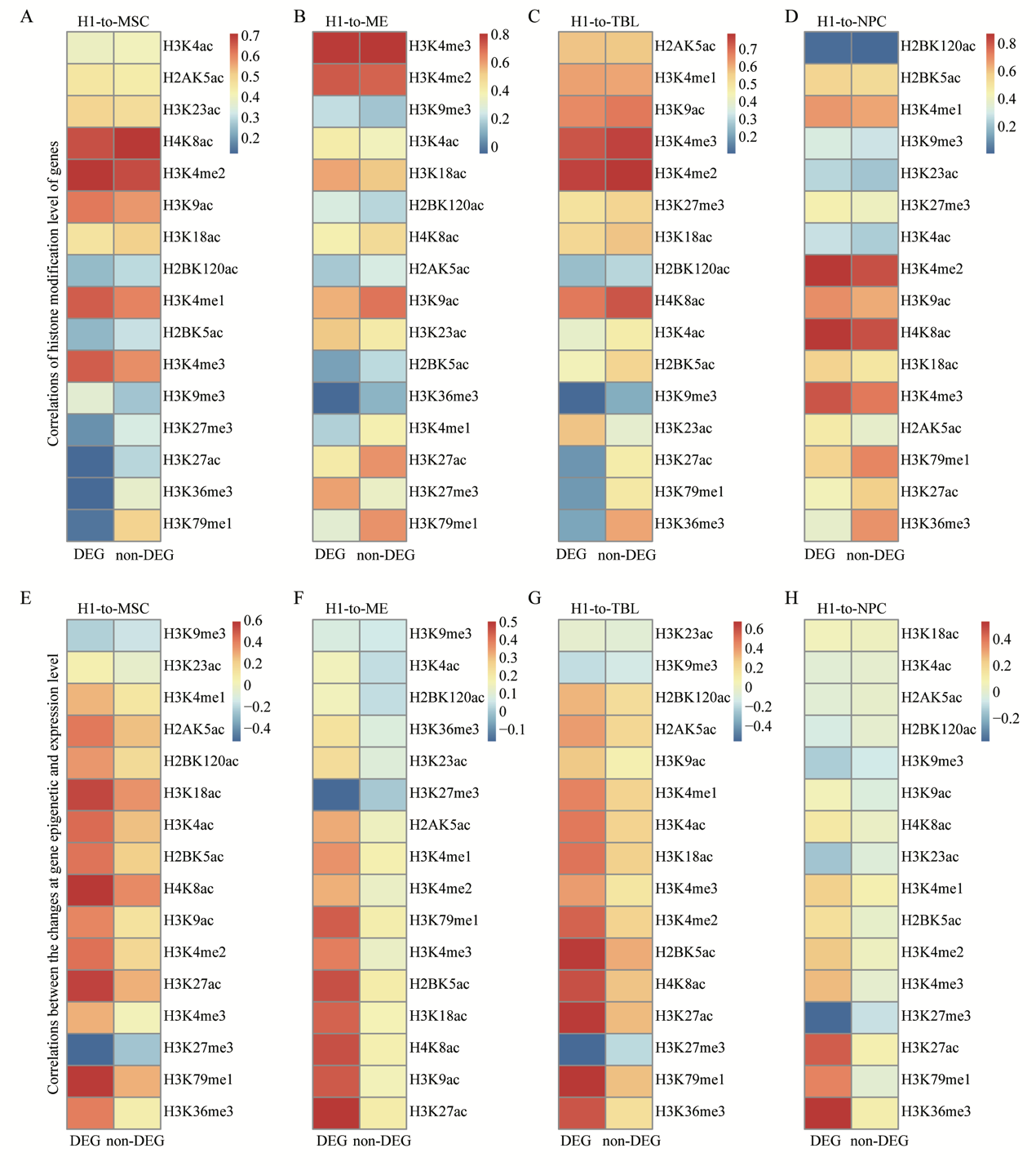


**Figure S7** (**A-D**) Spearman correlations in terms of the histone modification levels between H1 and MSC, between H1 and ME, between H1 and TBL, and between H1 and NPC, respectively, for DEGs and non-DEGs, respectively. (**E-H**) Spearman correlations in terms of the expression changes and epigenetic changes for each mark between H1 and MSC, between H1 and ME, between H1 and TBL, and between H1 and NPC, respectively, for DEG and non-DEGs. The similar method of creating figure 7A was used to calculate the expression changes and epigenetic changes for each mark for the DEGs and non-DEGs. The marks in (A-H) are positioned by the difference in the correlations between DEGs and non-DEGs in ascending order.


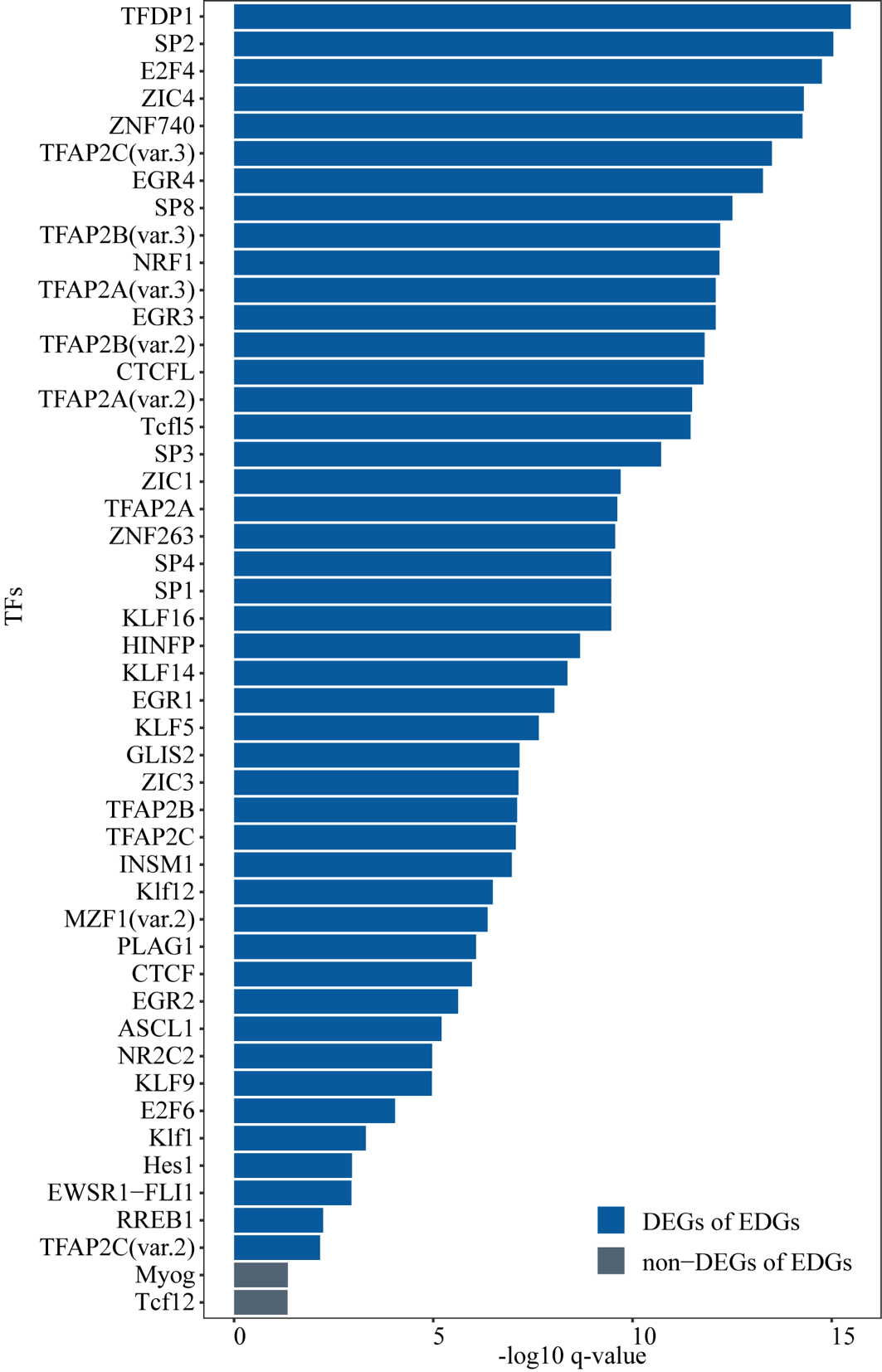


**Figure S8** TFs that preferentially bind to DEGs and non-DEGs among EDGs, respectively, between H1 and MSC. The bar length represents the significance level of q-value (in logarithmic scale).


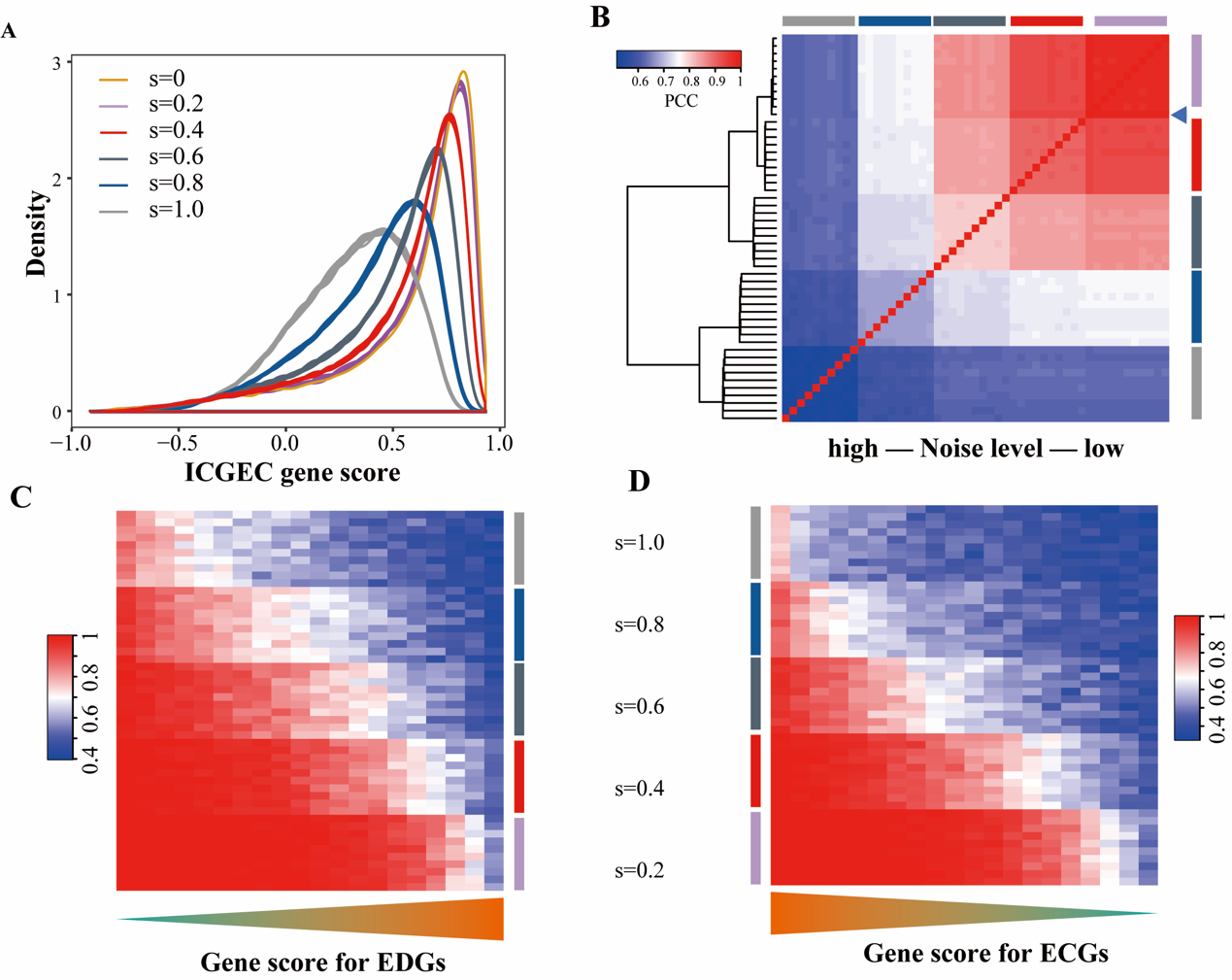


**Figure S9** Effect of additional noise on the performance of ICGEC. **(A)** Density plot showing the shift of the gene score produced from datasets without (s=0) or with (s=0.2, 0.4, 0.6, 0.8 and 1.0) additional artificial noise. **(B)** Hierarchical clustering plot showing the similarity of gene scores produced at different noise levels. The similarity is estimated as PCC over all genes with regard to their gene scores. The arrow indicates the dataset without additional noise. **(C-D)** Heatmaps showing the overlap degree of EDGs **(C)** and ECGs **(D)**, respectively, identified between using datasets with additional noise at five levels and without. The EDGs or ECGs identified at the five noise levels were divided into 20 bins of approximately equal size, then the number of genes that were present at the equivalent bins from the dataset without additional noise were counted. For this part of analysis, we began with the epigenetic data matrices (s=0) from the H1 and MSC cell lines, then we added different levels of additional noise to both matrices to produce artificially less perfect datasets, finally we evaluate the performance of ICGEC based on the results shown here. The added noise was derived from the distribution U (-1, 1) ×s ×e, where s represented the noise strength from low to high with corresponding values from 0.2 to 1 with a step size of 0.2, e was the original modification level, and U (-1,1) denoted a uniform distribution from -1 to 1. At each noise level, we produced 10 simulated datasets. Our results indicate that ICGEC performs well in despite of low-to-moderate level of noise.

**
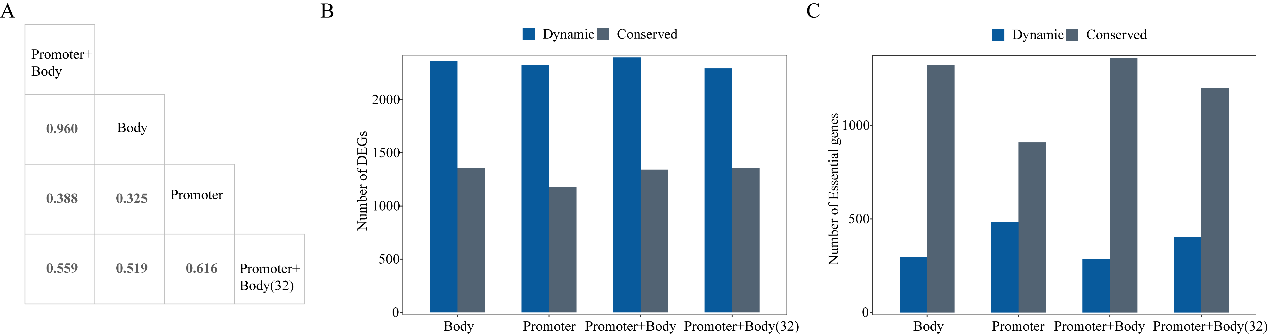
**

**Figure S10 Comparison of ICGEC using epigenetic signal data from four methods.** **(A)** Pair-wise Pearson correlation coefficient of ICGEC gene scores derived from using the four methods that quantify the epigenetic signal of genes from the promoter alone (Promoter), gene body regions alone (Body), from the promoter and gene body regions (Promoter+Body), and from the promoter and gene body regions, separately (Promoter+Body (32)). **(B, C)** Bar plot showing the number of DEGs (B) and essential genes (C) in EDGs or ECGs, respectively. The genes with ICGEC gene scores on the top and bottom one-quarter of all genes were defined as epigenetically dynamic genes (EDGs) and epigenetically conserved genes (ECGs), respectively.


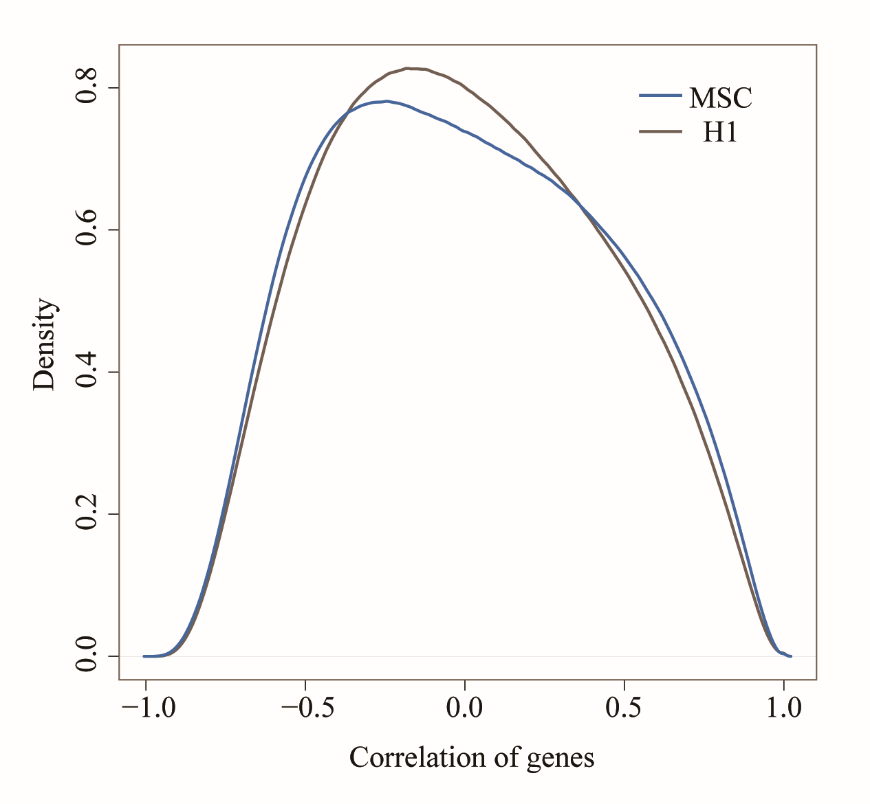


**Figure S11 Comparison of the distributions of gene-gene correlation coefficients between H1 and MSC cell lines.** Density plot showing the distribution pattern of gene-gene correlation coefficients, which were calculated from the gene epigenetic context matrices for H1 and MSC cell lines, separately. Kolmogorov-Smirnov test indicates that two distributions are significantly (P-value < 0.05) different from each other though the two density plots seem to be similar to some extent.
